# Modulating CRISPR gene drive activity through nucleocytoplasmic localization of Cas9 in *S. cerevisiae*

**DOI:** 10.1186/s40694-019-0065-x

**Published:** 2019-02-04

**Authors:** Megan E. Goeckel, Erianna M. Basgall, Isabel C. Lewis, Samantha C. Goetting, Yao Yan, Megan Halloran, Gregory C. Finnigan

**Affiliations:** 1 Department of Biochemistry and Molecular Biophysics, 141 Chalmers Hall, Kansas State University, Manhattan, KS 66506 USA; 2 Present Address: Department of Psychology, 106-B Kastle Hall, University of Kentucky, Lexington, KY 40506 USA

**Keywords:** CRISPR, Cas9, Gene drive, Biotechnology, Nucleocytoplasmic trafficking, Yeast

## Abstract

**Background:**

The bacterial CRISPR/Cas genome editing system has provided a major breakthrough in molecular biology. One use of this technology is within a nuclease-based gene drive. This type of system can install a genetic element within a population at unnatural rates. Combatting of vector-borne diseases carried by metazoans could benefit from a delivery system that bypasses traditional Mendelian laws of segregation. Recently, laboratory studies in fungi, insects, and even mice, have demonstrated successful propagation of CRISPR gene drives and the potential utility of this type of mechanism. However, current gene drives still face challenges including evolved resistance, containment, and the consequences of application in wild populations. Additional research into molecular mechanisms that would allow for control, titration, and inhibition of drive systems is needed.

**Results:**

In this study, we use artificial gene drives in budding yeast to explore mechanisms to modulate nuclease activity of Cas9 through its nucleocytoplasmic localization. We examine non-native nuclear localization sequences (both NLS and NES) on Cas9 fusion proteins in vivo through fluorescence microscopy and genomic editing. Our results demonstrate that mutational substitutions to nuclear signals and combinatorial fusions can both modulate the level of gene drive activity within a population of cells.

**Conclusions:**

These findings have implications for control of traditional nuclease-dependent editing and use of gene drive systems within other organisms. For instance, initiation of a nuclear export mechanism to Cas9 could serve as a molecular safeguard within an active gene drive to reduce or eliminate editing.

**Electronic supplementary material:**

The online version of this article (10.1186/s40694-019-0065-x) contains supplementary material, which is available to authorized users.

## Background

Control of biological populations is critical to agriculture, ecological conservation, and human health. Numerous methods have been employed to remove invasive species [1, 2], crop-damaging pests [3–6], or metazoans that harbor diseases [7, 8] including physical barriers, chemical agents, and/or natural predators or competitors. However, the ability to genetically modify an entire species has been hindered by the natural laws of segregation—introduction of a genetic element through natural breeding would require an unattainable number of modified individuals to be released into the wild. Given the introduction of CRISPR/Cas9 as an efficient, convenient, and universal genome editor [9–15], a mechanism has been developed that is *Super*-Mendelian in nature: a nuclease “gene drive.”

This arrangement of the CRISPR components is simple in design, yet powerful in application. The basic architecture includes a nuclease of choice (usually *S. pyogenes* Cas9, although many alternatives and engineered variants now exist) and the corresponding single guide RNA (sgRNA) expression cassette integrated within the genome. Placement of Cas9/sgRNA could be at a safe harbor locus or could delete or disrupt an existing endogenous gene. In the case of the former, the gene drive (GD) would likely also contain a “cargo” element—the intended genetic element to be delivered to the entire population. This could include any number of variations including endogenous or exogenous DNA to modify the organism itself (e.g. imposed fitness cost) or to aid in the separation between the host and disease-causing agent. Once expressed, the nuclease is primed by the guide RNA to target the *wild*-*type* copy of the gene (or position) on the homologous chromosome within a diploid genome (within the progeny between a gene drive individual and a wild-type individual) to create a double strand break (DSB). The unique arrangement of the GD relative to the DSB allows the expression cassette for Cas9/sgRNA itself to serve as the donor DNA for homology directed repair (HDR). The GD copies itself to the wild-type chromosome to repair the break and replaces the entire endogenous locus; a heterozygous cell (GD/WT) becomes a *homozygous* (GD/GD) cell. Action of a gene drive within a population would allow the rapid “forced” propagation of any genetic element in a small number of generations and would require only a small number of released GD individuals.

There are numerous applications of gene drive biotechnology to control and alter biological populations including global challenges such as eliminating insect-borne diseases [16–18]. Recent experimental [19–24] and computational studies [25–27] highlight the potential of GD systems. However, there remain many unknowns surrounding implementation and management of this new technology (including accidental or malicious release of such a system without any safeguard or inhibitory mechanism). Release of a GD-organism has the potential to modify a portion of the natural population of the chosen species, even using the current available gene drives (for which GD-resistance is still an ongoing issue) [28]. Therefore, it is critical to identify means to control, titrate, inhibit, or reverse gene drive systems to modulate or slow their progression, and as a failsafe should removal of GD individuals become necessary.

Our previous work focused on examination of conserved components of CRISPR gene drives (e.g. nuclease, guide RNA, DNA repair) in budding yeast to identify modes of control, regulation, and inhibition of drive success in vivo [29, 30]. A variety of molecular mechanisms have been shown to modulate Cas9-based editing including nuclease expression, guide RNA sequence, Cas9–dCas9 fusions, anti-CRISPR mutants, and nucleocytoplasmic shuttling of tagged Cas9. Here, we expanded upon our previous work [29] (which focused on utilizing the SV40 signal) to examine additional NLS and NES combinations appended to *S. pyogenes* Cas9–eGFP fusion constructs within an artificial GD system. We tested three monopartite NLS sequences, mutated signals, and two NES signals to demonstrate titration of gene drive activity in a diploid yeast model.

## Results

### Non-native nuclear localization signals can direct Cas9 localization in vivo

Nucleocytoplasmic transport of macromolecules within eukaryotic cells is highly conserved [31–35] and has been recognized as a universal requirement of gene editing in living systems—namely, the intended nuclease must gain access to the interior of the nucleus and genomic content. This intracellular trafficking system involves recognition of nuclear import sequences by karyopherins for transit through the nuclear pore complex [36–38]. Use of the CRISPR/Cas system for alteration of the genome typically includes appending one or more NLSs to the nuclease (e.g. Cas9) and the classical NLS^SV40^ is often used for this purpose [10, 39, 40]. However, several groups have demonstrated that alteration of the nuclear localization of Cas9 can serve as a means to control editing. For instance, the iCas system was constructed to prevent nuclear entry until addition of an external cue [41]. Moreover, design of a split Cas9 included use of a NES sequence to restrict localization of one of the two halves of the nuclease until addition of an exogenous signal [42]. Finally, optimization of CRISPR-based editing in various organisms and cell types has focused on the placement, number, and identity of the included NLS sequence—whether native or non-native to the species of interest [43–49].

Our previous work in budding yeast demonstrated that the dynamic localization for Cas9 fusions harboring both NLS and NES signals resulted in a variable level of genomic editing (both in haploid and diploid cells) [29]. However, this work focused exclusively on the commonly used NLS^SV40^ signal. A previous study [50] demonstrated that mutational substitutions to a set of artificially derived NLS signals (from random peptide libraries) allowed for a spectrum of nuclear import efficiencies using a GFP-based reporter system. We sought to test whether alternative nuclear signals could still direct Cas9 to the nucleus and allow for a titration of DSB formation in a CRISPR gene drive diploid strain.

**Fig. 1:**
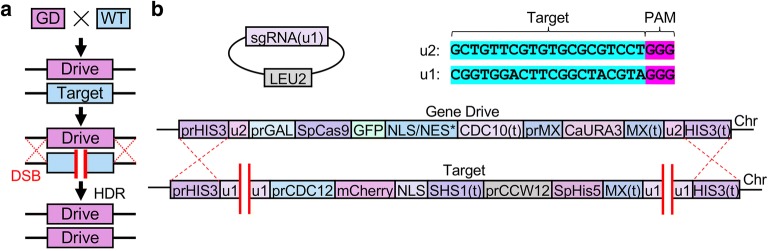
Design of an artificial CRISPR gene drive system in *Saccharomyces cerevisiae*. **a** General schematic of a gene drive in a diploid genome. **b** An artificial gene drive system was constructed at the yeast *HIS3* locus. The inducible *GAL1/10* promoter drives expression of a codon-optimized *S. pyogenes* Cas9 containing a C-terminal eGFP fusion followed by a chosen nuclear signal sequence (NLS/NES* see Fig. 2a). An inserted terminator (from *CDC10*) was placed downstream of the Cas9 coding sequence followed by a selectable marker cassette. This included the non-native MX-based promoter and terminator sequences driving constitutive expression of the *C. albicans URA3* gene. The entire gene drive system was flanked by two identical artificial sites, termed (u2), that do not exist in the native genome [29, 51]. The design of the drive also included an engineered “target” cassette (*bottom*) built within a strain of the opposite mating type at the *HIS3* locus. This included an artificial “cargo” gene and a yeast-based terminator (from *SHS1*). A modified selectable marker cassette included the constitutive *CCW12* (cell wall) promoter sequence driving the *S. pombe HIS5* gene (functional equivalent to yeast *HIS3*). Finally, two (u1) sequences were inserted flanking the entire target locus. To complete action of the drive, a high-copy plasmid (pGF-V1220) contained the cassette for the guide RNA (marked with *LEU2*)

**Fig. 2:**
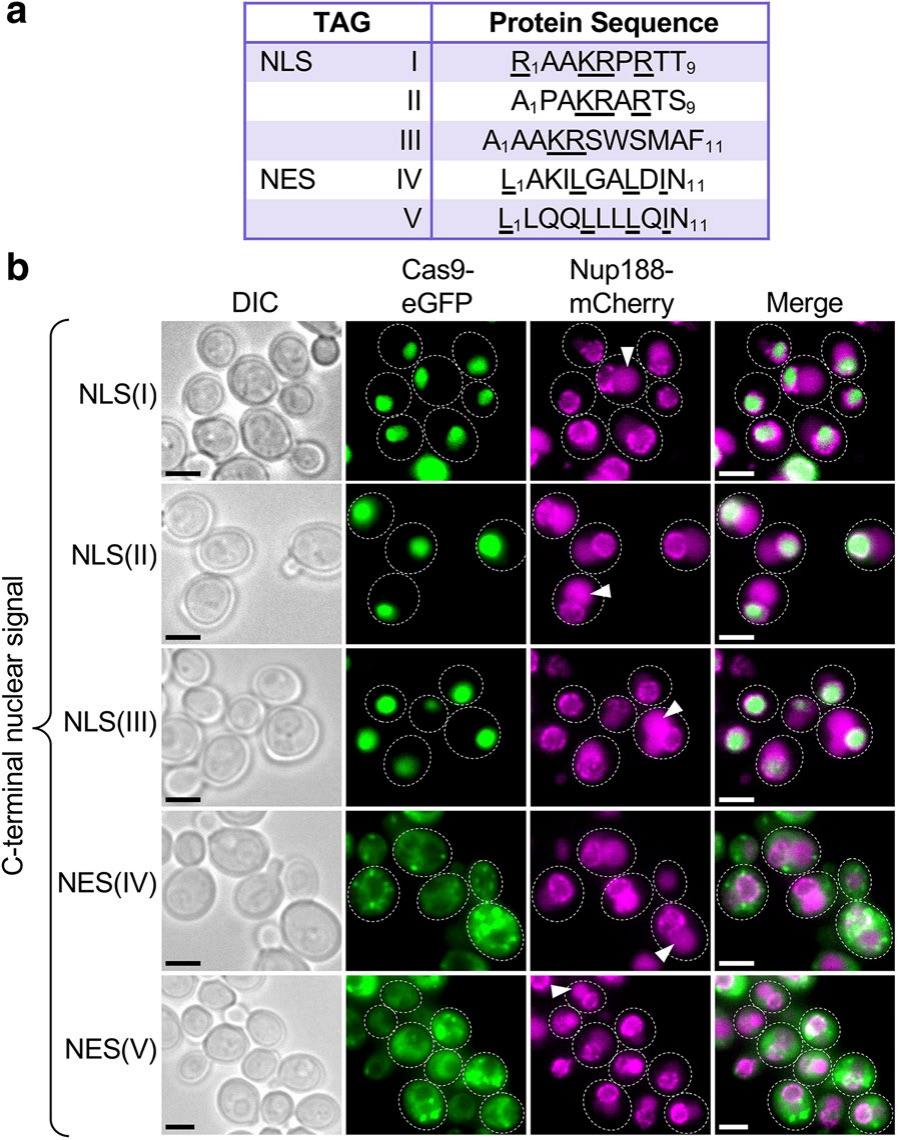
Subcellular localization of *S. pyogenes* Cas9 tagged with various nuclear localization sequences. **a** Table of non-native NLS and NES sequences tested; basic residues are underlined for NLSs and hydrophobic residues are underlined for NESs. **b** Fluorescence microscopy of live yeast cells containing NLS sequences (GFY-3435 to 3437) or NES sequences (GFY-3438, 3439) fused to eGFP-tagged Cas9 (also see Table 1). Yeast were cultured in galactose prior to imaging. An integrated copy of Nup188-mCherry marked the nuclear periphery. Representative images are shown; white dotted lines, outline of selected cells. Scale bar, 3 μm. Triangles indicate the yeast vacuole

Design of our gene drive system allows for the safe and programmable examination of various CRISPR components. Briefly, two sets of “unique” Cas9 target sites (termed u1 and u2) [51] are positioned flanking both the inducible Cas9-expression cassette (“drive”) and the corresponding locus harboring a selectable marker (“target”). Activation of Cas9 and inclusion of the correct sgRNA fragment (expressed from a plasmid) would cause multiplexing and cleavage of the dual (u1) sites in the target chromosome and repair via homologous recombination using the drive-containing chromosome as the source of donor DNA (Fig. 1). Haploid yeast strains harboring an inducible Cas9–eGFP construct were generated using various C-terminal signals: NLS signals included the classical SV40 and three other monopartite signals with sequences containing either four (NLS-I), three (NLS-II), or two (NLS-III) lysine and/or arginine residues within the motif whereas the export signals (NES-IV and NES-V) were derived from the ΦX_3_ΦX_2_ΦXΦ [52] consensus (Fig. 2a). These NLS signals were selected from a previous study [50]: they represented a diverse set of artificially derived peptides found to direct nuclear import across different cellular systems—we chose several that (1) varied in the number and placement of basic residues and (2) had a number of residues within the signal that, when mutated, were found to generate a spectrum of activities through an in vivo cellular assay. Following Cas9 induction in media containing galactose, strains were imaged by fluorescence microscopy (Fig. 2b). Localization was determined by labeling the nuclear periphery with a mCherry-marked integrated copy of Nup188, a nuclear pore complex component. For all three non-native NLSs, Cas9 localized to the nucleus and for both NES-tagged Cas9 fusions, eGFP signal was occluded from the nucleus. However, despite the steady-state localization of the Cas9–eGFP–NES constructs, our previous findings suggested that a small amount of editing (and gene drive activity in diploids) does take place, albeit after significantly longer nuclease induction times [29].

### Mutational alterations to NLS or NES sequences can modulate gene drive activity

**Fig. 3:**
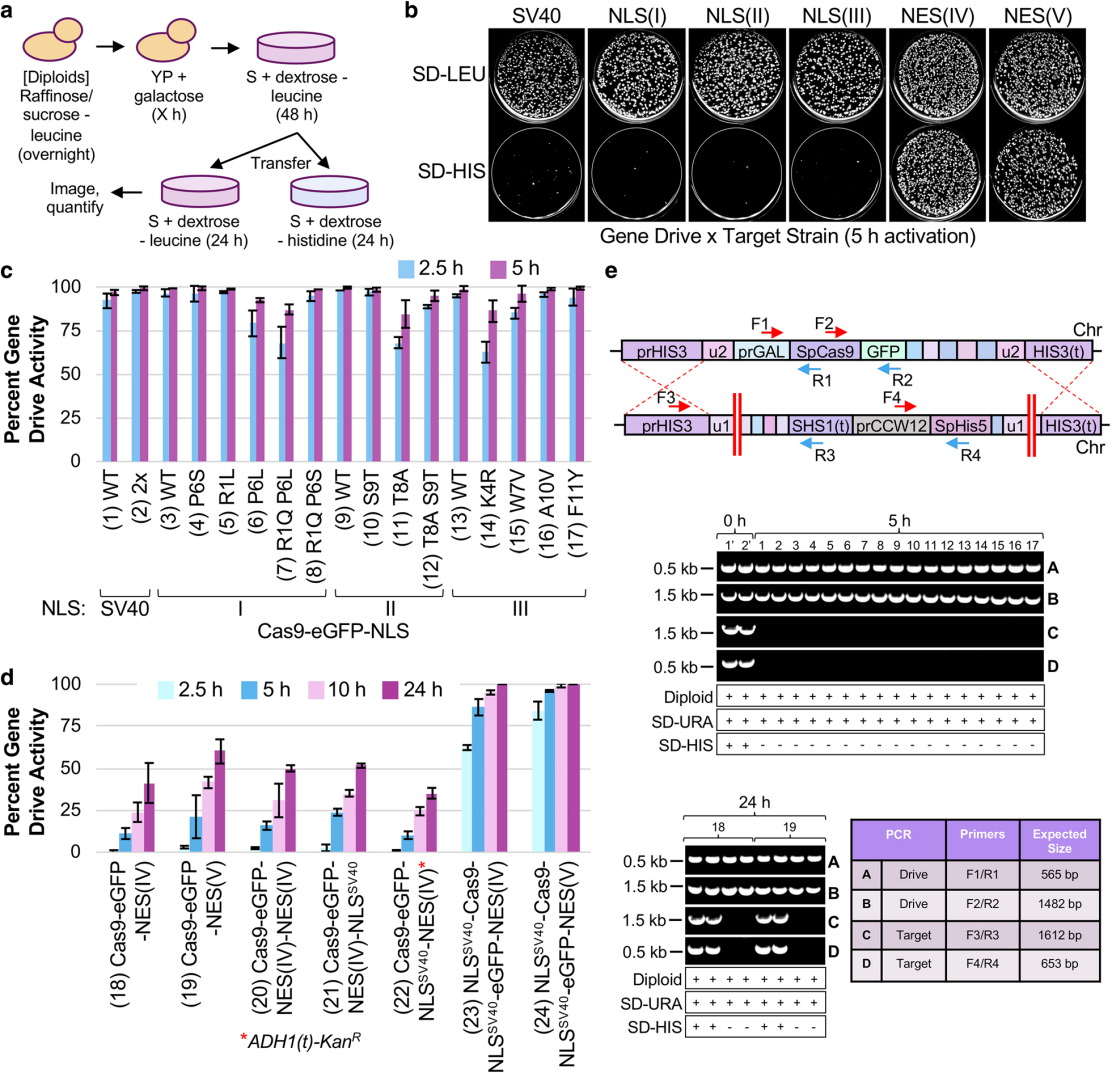
CRISPR gene drives using various NLS and NES fused to Cas9–eGFP. **b** Schematic of gene drive activation. Following diploid selection, yeast were grown to saturation overnight in media containing raffinose and sucrose lacking leucine. Cultures were back-diluted into rich medium containing galactose for a set number of hours, diluted to approximately 100–500 cells per agar plate (SD-LEU), and incubated for 48 h. Yeast colonies were velvet-transferred to SD-LEU and SD-HIS plates for up to 24 h before imaging. If the GD was successful and removed the target *HIS3* locus (harboring *SpHIS5*), then colonies would be sensitive to the SD-HIS condition. **b** Haploid yeast strains (GFY-2756, and GFY-3465-3469) were mated to target strains (GFY-3206 and 3207), diploids selected, and gene drives activated for 5 h. Yeast were plated on SD-LEU and transferred to a final SD-LEU plate (control) and SD-HIS plate to assess gene drive activity. **c** The number of colonies sensitive on SD-HIS provided a measure for “percent gene drive activity”. Diploid gene drives were tested using strains from **b** and mutational substitutions made to each NLS (GFY-3470, 3443-3447, 3449-3452, and 3454-3456, numbered 1–17) where Cas9 was induced for either 2.5 h or 5 h and quantified for drive activity. Error, SD. NLS(I–III) sequences can be found in Fig. 2a. **d** Gene drive strains (GFY-3468, 3469, 3471, 3472, 2758, 3716 and 3717) harboring a NES signal in the absence or presence of additional NLSs were tested for 2.5 h, 5 h, 10 h, and 24 h of Cas9 induction and quantified as in (**c**). Error, SD. Red asterisk, this construct harbors the *ADH1(t)*-*prMX*-*Kan*^*R*^-*MX(t)* cassette following Cas9–eGFP. **e** *Top*, illustration of the gene drive/target arrangement and the position of oligonucleotides (Additional file 1: Table S1) used. *Middle*, PCRs were performed on chromosomal DNA from clonal isolates from each drive (5 h). The numbers (1–17) correspond to strains from Fig. 3c. The expected sizes for each PCR **a**–**d** are shown along with markers. Images were cropped from independent gels or portions of larger gels and are divided by white lines. Two isolates were obtained with no galactose activation (1’ and 2’; dextrose only treatment) from GFY-2756 (Strain 1). All colonies were tested for ploidy status (diploid) and growth on SD-URA (drive) and SD-HIS (target). Strain GFY-2756 was tested on G418 media. *Below*, A similar analysis of clonal isolates from the NES-containing strains was performed (24 h). Two isolates each (from strain 18 and 19) were chosen that were either resistant or sensitive to the SD-HIS condition

We tested each NLS and NES sequence appended to Cas9–eGFP for its effect on gene drive activity in diploid yeast. The general methodology employed to assay gene drive function in vivo included a variable induction time in galactose followed by recovery on dextrose-containing media (Fig. 3a). Following the formation of single colonies, yeast were transferred to medium lacking histidine to test for the presence of the *SpHIS5* marker—equivalent to yeast *HIS3*—within the target strain. Upon successful action of the drive, the entire target locus is removed, and colonies are unable to grow in the absence of histidine (e.g. SD-HIS plates). Importantly, our artificial system does not impose any selection for or against action of the gene drive unlike other possible arrangements that challenge cells by selecting for successful DSB/repair events. Compared to the NLS^SV40^-tagged strain, all three artificial NLS signals (I–III) caused a dramatic loss of growth on SD-HIS; constructs harboring the NES (IV, V) retained a large number of viable colonies (Fig. 3b). Previous work suggested that mutations to positions along the length of these artificial signals altered their effectiveness at promoting nuclear import by a fluorescence reporter system in live cells [50]. Therefore, we generated twelve substitutions across NLS(I–III) and examined their gene drive activities in vivo (Fig. 3c). The total percentage of colonies sensitive to the SD-HIS condition was quantified across multiple trials. As predicted, a NLS^SV40^ tag and a tandem NLS^SV40^–NLS^SV40^ tag allowed for > 95% activity at 5 h of Cas9 induction (1, 2). To ensure that high-expression levels from a 5 h induction were not masking subtle effects of the alteration to the NLS signal, we also tested each strain at a lower 2.5 h induction. Many of the substitutions to other classes of NLS (I–III) displayed no change compared to the WT and only four of the twelve substitutions (6, 7, 11, and 14) had a modest decrease in activity. Therefore, these results demonstrate that some of the NLS alterations can partially reduce Cas9 editing in vivo to varying degrees.

The two NES signals tested on Cas9–eGFP included a PKI-like sequence [53] (NES-IV) as well as a similar NES with added leucine residues present within the motif (NES-V). A previous study reported that excessive hydrophobic amino acids could interfere with nuclear export signals [52]. Therefore, we tested a NES harboring six total leucine residues as opposed to only three in the PKI-like motif (Fig. 2a)—the expectation was that a reduction in NES effectiveness would manifest in higher nuclear residence time and gene drive efficiency. Our previous work demonstrated that a significantly longer (24 h) induction time was required to observe drive activity for a construct harboring only a single NES signal [29]; to allow for a comparison to the NLS-containing strains, we chose to treat cells in galactose for 2.5, 5, 10, and 24 h (Fig. 3d). For induction times less than 10 h, the gene drive activity (18, 19) remained below 50% (Fig. 3d). We noticed a subtle difference between the effectiveness of the two NES-containing constructs and it appeared that the original PKI-like motif (18) provided less drive activity across multiple time points compared to the sequences with added leucine residues (19). A tandem NES^(PKI-like)^–NES^(PKI-like)^ signal (20) did not provide any significant change compared to a single NES alone. Previously, we found that a C-terminal fusion of the dual NLS^(SV40)^–NES^(PKI-like)^ sequence to Cas9–eGFP (22) seemed to phenocopy a fusion of NES^(PKI-like)^ alone [29]. Here, we constructed and tested the reciprocal fusion, NES^(PKI-like)^–NLS^(SV40)^ (21), to determine its effect in vivo. We observed that both constructs displayed similar gene drive activities to a single NES signal, yet positioning of the NLS^SV40^ on the extreme C-terminus appeared to provide slightly higher activity (and less contribution from the NES). Finally, we included both NES signals in a construct also harboring two additional NLS^SV40^ sequences—at the N-terminus and fused between Cas9 and eGFP (23, 24). The competition between two NLS sequences versus one NES sequence provided a high level of gene drive activity, with a slight increase in activity from the construct harboring the leucine-rich NES (24). Together, these data illustrate that changes can also be made to either the primary sequence or placement of nuclear export signals to shift the level of editing in vivo.

Previous work has demonstrated that gene drives may induce a DSB but fail to copy the GD cassette to the target chromosome. In such cases, NHEJ causes repair of the broken DNA ends and prevents HR-based propagation of the drive (e.g. GD “resistant” alleles). Therefore, to confirm that yeast sensitive to the SD-HIS condition had lost the target allele at the *HIS3* locus, we obtained clonal isolates from each gene drive experiment, tested for each of the included markers, assayed ploidy status, and examined purified genomic DNA by multiple diagnostic PCRs (Fig. 3e). For diploids lacking galactose treatment (0 h), four independent PCR reactions illustrated the presence of both the gene drive and target constructs. However, for isolates subjected to galactose induction (5 h), only the two PCRs corresponding to the gene drive allele (PCR-A, PCR-B) were present; reactions for the target allele were unable to amplify the expected fragment (PCR-C, PCR-D) suggesting the diploid genome was now homozygous for the drive allele (1–17). A similar analysis was performed for eight clonal isolates from the NES strains (18, 19). However, we chose to sample two isolates that were sensitive and two samples resistant to the SD-HIS condition as there were still many colonies displaying each phenotype after 24 h (Additional file 1: Fig. S3). The expectation was that diploid colonies still growing on SD-HIS medium were still heterozygous for the drive and target alleles at the *HIS3* locus. Diagnostic PCRs illustrated that the target allele was still present for surviving colonies and absent for colonies sensitive to SD-HIS (Fig. 3e, *below*). Given that these NES-containing strains had 24 h of galactose induction, the lack of editing (and GD activity) likely results from active nuclear export of Cas9. Collectively, these results demonstrated a continuum of gene drive activity ranging up to 100% in our yeast model across all Cas9–eGFP fusions with both NLS and/or NES signals (Fig. 3).

## Discussion

Given that application of the CRISPR/Cas editing biotechnology in eukaryotic systems requires delivery of Cas9/sgRNA to the nucleus, we focused on methodologies that could provide a new suite of molecular tools to control, inhibit, or modulate gene drive systems in vivo, although we recognize these techniques might be used for many types of genomic editing and could apply to alternative uses of the CRISPR system (e.g. dCas9). While there are still few studies on gene drives, the power and potential application for this technology is clear, despite the current challenges and obstacles. The ability to modify an entire population with a genetic element of choice presents numerous advantages including optimizing agricultural crops and animals, prevention of human disease, and ecological control on a large scale. However, ongoing testing of optimal designs including safeguards and controllable drives warrant further research and recent progress has been made in current systems [25, 26, 54–56]. Previous efforts (both computational and experimental) have highlighted a variety of components that might serve as a platform for control or inhibition of gene drives [21, 26, 29, 30]. Here, we focused on altering the nucleocytoplasmic localization of Cas9 for titration of editing.

While a popular choice for nuclear import across model systems has been the monopartite NLS^SV40^ signal, others have found that varying the position, copy number, or identity of the NLS (native or non-native) can alter genomic editing [43, 46, 48, 57, 58]. Given the conservation of nuclear import machinery across eukaryotes and the wide variety of possible nuclear import sequences (native or artificial), this could present a complex platform for tuning or optimizing Cas9 nuclear import in any species of interest. As we have demonstrated here, three artificially generated NLS signals allowed for efficient nuclear entry and subsequent gene drive activity in vivo; also, some mutational substitutions to the NLS primary sequence partially reduced editing. Future iterations might include modifications to signal positioning (N- or C-terminus, or between fusion proteins), the distance from Cas9, and local residue context surrounding the signal.

Previous studies have evaluated the sequence composition of many natural and artificial NLS and NES sequences through mutagenesis [59], in vivo subcellular localization assays [50, 52], in vitro binding experiments [60], and computational analyses [61–63]. Consensus sequences have been developed for different classes of NLS (for example, the K–(K/R)–X–(K/R) monopartite signal) that highlight critical residues within the signal. However, there is also a contribution of flanking amino acids (not part of the consensus) that are upstream, downstream, or within the NLS itself [59, 64–66]. For NESs, multiple classes of signals have now been defined that are variations of the Φ–X_3_–Φ–X_2_–Φ–X–Φ consensus. Previous work has highlighted further preferences and restrictions to NES motifs and these may also be context-dependent within three-dimensional protein structures [52, 61, 62]. For instance, the presence of certain residues (such as proline) may disrupt NESs [52]. Comparison of our NES(IV) versus NES(V) illustrated that the addition of hydrophobic residues (leucine) was less efficient as an export signal, similar to previous findings [52]. Given the large variation in nuclear signals, the contribution of individual residues to NLS or NES function will require experimental validation. Furthermore, the assays used to detect nuclear import and/or export need to be taken into consideration. In the study performed by Kosugi et al. [50], a GFP reporter system in live cells was used to quantify the success of nuclear import. In our gene drive experiments, we assayed the effect of Cas9-dependent DSB formation followed by DNA repair and a resulting growth phenotype. Furthermore, interpretation of nuclear entry or exit may be complicated by additional cryptic signals (enhancement or competition with the signal(s) of interest) or protein size and entry through the nuclear pore complex.

Of note, our data demonstrates that inclusion of a NES (even when not paired with any NLS signal) still allowed for some level of editing as Cas9 may gain entry to the nucleus through diffusion followed by export; editing without any appended NLS has also been observed in previous studies [29, 43]. Therefore, we recommend use of various nuclear signal combinations to modulate and reduce, rather than eliminate, editing. However, nuclear restriction of Cas9 may still be useful when paired with other inhibitory mechanisms such as the AcrIIA2 and AcrIIA4 anti-CRISPR proteins [30], reduced or programmed expression of nuclease transcript [29], or other mechanisms to inhibit editing such as degradation of Cas9 [67].

Restriction of Cas9 nuclear localization has also been demonstrated in other cell types through (1) occlusion of a NLS signal or (2) tethering to the plasma membrane—subsequent release was achieved by activation of a protease to cleave the tethered dCas9–NLS construct and allow transport into the nucleus [41, 68, 69]. However, our study provides experimental evidence for use of this general methodology for titration of gene drive activity. We envision that nuclear occlusion of Cas9 could also be achieved by alternative approaches. Expression of an inducible anti-GFP “nanobody”-containing peptide fused with an export signal might provide temporal control to regulate Cas9–GFP nucleocytoplasmic localization. In this way, activity might be reduced at a later point in time (or to a specific portion of a population) by causing an increase in Cas9 nuclear export. A secondary system that could modulate Cas9 activity—inducible by external stimuli—would provide a suite of new options for controlling gene drive propagation within a population. This could be utilized as a molecular safeguard to slow or inhibit drives, allow for the timely use of anti-drives or other countermeasures, or as a means to cycle gene drive-containing organisms with seasonal or environmental changes. Alternatively, modification of the nuclear pore complex might allow for selective entry of a pool of Cas9 fusion constructs while restricting other variants or orthologs.

## Conclusion

In this study, we expanded our analysis of nuclear trafficking to control Cas9 editing within the context of a gene drive system. We predict that a combinatorial approach of altering nuclease (transcript and/or protein) levels, NLS/NES signals, cellular traps, and other inducible/tunable systems might be employed in the design of future gene drive systems that are safe, controllable, and/or reversible.

## Materials and methods

### Yeast strains and plasmids

*Saccharomyces cerevisiae* strains used in this study can be found in Table 1. Molecular biology techniques were used to generate all engineered constructs [70]. The general strategy included first constructing a *CEN*-based plasmid using in vivo assembly in yeast [71] using a modified lithium acetate transformation protocol [72]. Next, PCR amplified DNA of the entire assembled cassette, followed by treatment with *DpnI* enzyme (to remove template DNA), was integrated into the appropriate haploid yeast genome (for Cas9, the *HIS3* locus). For generation of NLS substitutions, a modified PCR mutagenesis protocol was used [73]. To integrate various eGFP–NLS or eGFP–NES combinations, a second integration construct was generated; universal eGFP and *MX(t)* sequences were used for homologous recombination (Table 1). Finally, selection markers were converted using a third round of integration (e.g. from *SpHIS5* to *CaURA3*) using common *CDC10(t)* and the *MX(t)* sequences. The only plasmid used in this study was the high-copy pRS425-based sgRNA(u1) vector (pGF-V1220) [29]. Diagnostic PCRs and Sanger DNA sequencing of all chromosomal modifications was performed to confirm successful integration events (see Additional file 1: Fig. S1).

**Table 1:** Yeast strains used in this study

Strain	Genotype	References
BY4741	*MAT* ***a*** *his3∆1 leu2∆0 met15∆0 ura3∆0*	[74]
BY4742	*MATα his3∆1 leu2∆0 lys2∆0 ura3∆0*	[74]
GFY-3206^a^	*BY4742; his3Δ::(u1)::prCDC12::mCherry::NLS* ^*SV40*^ *::SHS1(t)::prCCW12::SpHIS5::MX(t)::(u1)::HIS3(t)*	[29]
GFY-3207	*BY4742; his3Δ::(u1)::prCDC12::mCherry::SHS1(t)::prCCW12::SpHIS5::MX(t)::(u1)::HIS3(t)*	[29]
GFY-2756^b^	*BY4741; his3Δ::(u2)::prGAL::SpCas9::eGFP::NLS* ^*SV40*^ *::ADH1(t)::prMX::Kan* ^*R*^ *::MX(t)::(u2)::HIS3(t)*	[29]
GFY-3470^c^	*BY4741; his3Δ::(u2)::prGAL::SpCas9::eGFP::NLS* ^*SV40*^ *::NLS* ^*SV40*^ *::CDC10(t)::prMX::CaURA3::MX(t)::(u2)::HIS3(t)*	This study
GFY-3465^d^	*BY4741; his3Δ::(u2)::prGAL::SpCas9::eGFP::NLS* ^*Class2*- *I*^ *::CDC10(t)::prMX::CaURA3::MX(t)::(u2)::HIS3(t)*	This study
GFY-3443^e^	*BY4741; his3Δ::(u2)::prGAL::SpCas9::eGFP::NLS(P6S)* ^*Class2*- *I*^ *::CDC10(t)::prMX::CaURA3::MX(t)::(u2)::HIS3(t)*	This study
GFY-3444	*BY4741; his3Δ::(u2)::prGAL::SpCas9::eGFP::NLS(R1L)* ^*Class2*- *I*^ *::CDC10(t)::prMX::CaURA3::MX(t)::(u2)::HIS3(t)*	This study
GFY-3445	*BY4741; his3Δ::(u2)::prGAL::SpCas9::eGFP::NLS(P6L)* ^*Class2*- *I*^ *::CDC10(t)::prMX::CaURA3::MX(t)::(u2)::HIS3(t)*	This study
GFY-3446	*BY4741; his3Δ::(u2)::prGAL::SpCas9::eGFP::NLS(R1Q/P6L)* ^*Class2*- *I*^ *::CDC10(t)::prMX::CaURA3::MX(t)::(u2)::HIS3(t)*	This study
GFY-3447	*BY4741; his3Δ::(u2)::prGAL::SpCas9::eGFP::NLS(R1Q/P6S)* ^*Class2*- *I*^ *::CDC10(t)::prMX::CaURA3::MX(t)::(u2)::HIS3(t)*	This study
GFY-3466^d^	*BY4741; his3Δ::(u2)::prGAL::SpCas9::eGFP::NLS* ^*Class2*- *II*^ *::CDC10(t)::prMX::CaURA3::MX(t)::(u2)::HIS3(t)*	This study
GFY-3449	*BY4741; his3Δ::(u2)::prGAL::SpCas9::eGFP::NLS(S9T)* ^*Class2*- *II*^ *::CDC10(t)::prMX::CaURA3::MX(t)::(u2)::HIS3(t)*	This study
GFY-3450	*BY4741; his3Δ::(u2)::prGAL::SpCas9::eGFP::NLS(T8A)* ^*Class2*- *II*^ *::CDC10(t)::prMX::CaURA3::MX(t)::(u2)::HIS3(t)*	This study
GFY-3451	*BY4741; his3Δ::(u2)::prGAL::SpCas9::eGFP::NLS(T8A/S9T)* ^*Class2*- *II*^ *::CDC10(t)::prMX::CaURA3::MX(t)::(u2)::HIS3(t)*	This study
GFY-3467^f^	*BY4741; his3Δ::(u2)::prGAL::SpCas9::eGFP::NLS* ^*Class3*^ *::CDC10(t)::prMX::CaURA3::MX(t)::(u2)::HIS3(t)*	This study
GFY-3452	*BY4741; his3Δ::(u2)::prGAL::SpCas9::eGFP::NLS(K4R)* ^*Class3*^ *::CDC10(t)::prMX::CaURA3::MX(t)::(u2)::HIS3(t)*	This study
GFY-3454	*BY4741; his3Δ::(u2)::prGAL::SpCas9::eGFP::NLS(W7V)* ^*Class3*^ *::CDC10(t)::prMX::CaURA3::MX(t)::(u2)::HIS3(t)*	This study
GFY-3455	*BY4741; his3Δ::(u2)::prGAL::SpCas9::eGFP::NLS(A10V)* ^*Class3*^ *::CDC10(t)::prMX::CaURA3::MX(t)::(u2)::HIS3(t)*	This study
GFY-3456	*BY4741; his3Δ::(u2)::prGAL::SpCas9::eGFP::NLS(F11Y)* ^*Class3*^ *::CDC10(t)::prMX::CaURA3::MX(t)::(u2)::HIS3(t)*	This study
GFY-3468^g^	*BY4741; his3Δ::(u2)::prGAL::SpCas9::eGFP::NES* ^*PKI*- *like*^ *::CDC10(t)::prMX::CaURA3::MX(t)::(u2)::HIS3(t)*	This study
GFY-3469^h^	*BY4741; his3Δ::(u2)::prGAL::SpCas9::eGFP::NES* ^*Consensus*^ *::CDC10(t)::prMX::CaURA3::MX(t)::(u2)::HIS3(t)*	This study
GFY-3471	*BY4741; his3Δ::(u2)::prGAL::SpCas9::eGFP::NES* ^*PKI*- *like*^ *::NES* ^*PKI*- *like*^ *::CDC10(t)::prMX::CaURA3::MX(t)::(u2)::HIS3(t)*	This study
GFY-3472	*BY4741; his3Δ::(u2)::prGAL::SpCas9::eGFP::NES* ^*PKI*- *like*^ *::NLS* ^*SV40*^ *::CDC10(t)::prMX::CaURA3::MX(t)::(u2)::HIS3(t)*	This study
GFY-2758	*BY4741; his3Δ::(u2)::prGAL::SpCas9::eGFP:: NLS* ^*SV40*^ *::NES* ^*PKI*- *like*^ *::ADH1(t)::prMX::Kan* ^*R*^ *::MX(t)::(u2)::HIS3(t)*	[29]
GFY-3716^i^	*BY4741; his3Δ::(u2)::prGAL::NLS* ^*SV40*^ *SpCas9::NLS* ^*SV40*^ *::eGFP::NES* ^*PKI*- *like*^ *::CDC10(t)::prMX::Kan* ^*R*^ *::MX(t)::(u2)::HIS3(t)*	This study
GFY-3717	*BY4741; his3Δ::(u2)::prGAL::NLS* ^*SV40*^ *SpCas9::NLS* ^*SV40*^ *::eGFP::NES* ^*Consensus*^ *::CDC10(t)::prMX::Kan* ^*R*^ *::MX(t)::(u2)::HIS3(t)*	This study
GFY-3435^j^	*BY4741; his3Δ::(u2)::prGAL::SpCas9::eGFP::NLS* ^*Class2*- *I*^ *::CDC10(t)::prCCW12::SpHIS5::MX(t)::(u2)::HIS3(t) NUP188::mCherry::ADH1(t)::prMX::CaURA3::MX(t)*	This study
GFY-3436	*BY4741; his3Δ::(u2)::prGAL::SpCas9::eGFP::NLS* ^*Class2*- *II*^ *::CDC10(t)::prCCW12::SpHIS5::MX(t)::(u2)::HIS3(t) NUP188::mCherry::ADH1(t)::prMX::CaURA3::MX(t)*	This study
GFY-3437	*BY4741; his3Δ::(u2)::prGAL::SpCas9::eGFP::NLS* ^*Class3*^ *::CDC10(t)::prCCW12::SpHIS5::MX(t)::(u2)::HIS3(t) NUP188::mCherry::ADH1(t)::prMX::CaURA3::MX(t)*	This study
GFY-3438	*BY4741; his3Δ::(u2)::prGAL::SpCas9::eGFP::NES* ^*PKI*- *like*^ *::CDC10(t)::prCCW12::SpHIS5::MX(t)::(u2)::HIS3(t) NUP188::mCherry::ADH1(t)::prMX::CaURA3::MX(t)*	This study
GFY-3439	*BY4741; his3Δ::(u2)::prGAL::SpCas9::eGFP::NES* ^*Consensus*^ *::CDC10(t)::prCCW12::SpHIS5::MX(t)::(u2)::HIS3(t)NUP188::mCherry::ADH1(t)::prMX::CaURA3::MX(t)*	This study

^a^The SV40 nuclear localization signal was SRADPKKKRKV. The artificial (u1) sites have the sequence 5′ ATGA**CGGTGGACTTCGGCTACGTA**GGGCGATT 3′ where the bold is the 20 bp target and the PAM is underlined [51]. *SpHIS5* refers to *Schizosaccharomyces pombe HIS5* (the functional equivalent of *S. cerevisiae HIS3*)

^b^The (u2) sequence includes 5′ **GCTGTTCGTGTGCGCGTCCT**GGG 3′ [51]. *SpCas9* refers to *Streptococcus pyogenes* Cas9

^c^The cloning strategy to construct GFY-3470 (and also GFY-3443-3447, 3449-3452, 3454-3456, 3465-3469, and 3471-3472) included first creating a parental vector (pGF-IVL1444) using yeast in vivo plasmid assembly [71] containing *eGFP*-*SpeI(site)*-*CDC10(t)*-*prCCW12*-*SpHIS5*-*MX(t)* on pRS315. Second, custom genes were synthesized (Genscript, Piscataway, NJ) containing the 3′ most 180 bp of eGFP, a C-terminal NLS or NES signal, stop codon, and 191 bps of the 3′ UTR of *CDC10*. Third, substitutions were made to the NLS sequence using a modified PCR mutagenesis protocol [73]. Fourth, the NLS/NES sequence was inserted into pGF-IVL1444 using in vivo assembly. Fifth, the entire construct (from eGFP through the MX terminator) was amplified using a high-fidelity polymerase (KOD Hot-Start, EMD Millipore), digested with DpnI, transformed into a yeast strain harboring an integrated *prHIS3*-*(u2)*-*prGAL*-*SpCas9*-*eGFP*-*ADH1(t)*-*prMX*-*KanR*-*MX(t)*-*(u2)*-*HIS3(t)* (GFY-2755), and selected on SD-HIS. Sixth, a second round of integration was used to convert the *CDC10(t)*-*prCCW12*-*SpHIS5*-*MX(t)* marker to *CDC10(t)*-*prMX*-*CaURA3*-*MX(t)* using pGF-IVL1412 as a template. Note, for constructs harboring dual signals (e.g. NLS^SV40^–NLS^SV40^), two glycine residues were included between the two sequences. Integration of all constructs was confirmed by growth phenotype, diagnostic PCRs, and DNA sequencing

^d^A previous study identified a number of novel classes of monopartite NLS signals from a random peptide library screen [50]. These were designated as “Class 2” NLS signals with a general structure of RXXKRXR (Class 2-I) or KRXR (Class 2-II) and a full consensus sequence of (P/R)XXKR(ˆED)(K/R) where (^ED) is any residue except Asp or Glu. The full sequences for the sampled NLSs included RAAKRPRTT and APAKRARTS, respectively

^e^Mutations were chosen [50] for each of the classes of identified NLS signals

^f^The consensus sequence for Class 3 NLS signals [50] was determined as KRX(W/F/Y)XXAF. The signal used was AAAKRSWSMAF

^g^The prototypical NES^(PKI-like)^ signal sequence was slightly modified to yield a NES of LAKILGALDIN [52, 53]

^h^The consensus sequence ΦX_3_ΦX_2_ΦXΦ where Φ is a hydrophobic residue (L, I, V, M, F, W, C, T, or A) of a Class 1a NES signal as determined previously [52]. The sequence used was LLQQLLLLQIN

^i^Yeast strains GFY-3716 and GFY-3717 were constructed similar to GFY-3470 but were transformed to *prHIS3*-*(u2)*-*prGAL*-*NLS*^*SV40*^*SpCas9*-*NLS*^*SV40*^-*eGFP*-*ADH1(t)*-*Kan*^*R*^-*(u2)*-*HIS3(t)* yeast (GFY-2759). Following integration of the C-terminal tag along with the *SpHIS5* marker, a final switch was performed (using pGF-IVL1412) to include the *CDC10(t)* sequence along with the Kan^R^ marker

^j^The mCherry tag was appended to the C-terminus of *NUP188* by transforming an amplified fragment of *NUP188(CT)*-*mCherry*-*ADH1(t)*-*prMX*-*CaURA3*-*MX(t)*-*NUP188(t)* including 500 bp of flanking sequence (DNA from GFY-3347) to create GFY-3435 to 3439

### Culture conditions

Budding yeast were grown on solid agar medium or in liquid cultures; rich media, YPD (2% peptone, 1% yeast extract, 2% dextrose), or synthetic drop-out media (nitrogen base, amino acids, and ammonium sulfate) were used. Prior to galactose metabolism (2%), cultures were grown to saturation in medium containing 2% raffinose and 0.2% sucrose. All sugars were filter sterilized.

### Fluorescence microscopy

Haploid yeast cultures were grown to saturation in synthetic complete medium containing raffinose and sucrose overnight. Next, strains were back-diluted into rich medium containing galactose for 4.5 h at 30 °C and prepared on a glass slide [29]. Live cells were examined using a Leica DMI6000 fluorescence microscope (Leica Microsystems, Buffalo Grove, IL). A Leica DFC340 FX camera, 100 × lens, and fluorescence filters (Semrock, GFP- 4050B-LDKM-ZERO, mCherry-C-LDMK-ZERO) were used. Software for image capture and analysis included Leica Microsystems Application Suite and ImageJ (National Institute of Health). Images were taken using identical exposure times; representative cells were chosen for each image set and scaled together. Scale bars, 3 μm. “Merged” images do not contain any additional processing from the two combined channels.

### CRISPR gene drives in diploid yeast

Haploid yeast strains harboring the *SpCas9* gene drive construct were first transformed with the high-copy *LEU2*-based sgRNA(u1) plasmid (pGF-V1220) and propagated on SD-LEU plates [29]. Next, Cas9/sgRNA-containing haploids were mated to target yeast strains of the opposite mating type on rich media for 24 h and transferred to diploid selection plates (SD-LEU-HIS) for three consecutive stages of selection. Diploids were cultured overnight to saturation (raffinose/sucrose), grown in rich medium containing galactose for (0 to 24 h) to express Cas9, diluted, spread onto SD-LEU medium at a density of 200–500 cells per plate, and incubated at 30 °C for 48 h. Finally, colonies were replica-plated by velvet transfer to a second SD-LEU and SD-HIS plate for 18–24 h, imaged, and the total surviving colony number was quantified in a single-blind fashion (between 100 and 250 colonies counted for each condition). Gene drive and target genome status were interrogated by diagnostic PCRs (also see Additional file 1: Fig. S2, Table S1) on isolated chromosomal DNA (also confirmed as diploids [29]). Genetic safeguards to contain yeast gene drives included the use of artificial sequences [51] programmed at the *HIS3* locus (u1 sites for targeting), a self-excision module (u2 sites) flanking Cas9 constructs [29], an inducible promoter driving Cas9 expression (*prGAL1/10*), and sgRNA plasmids on an unstable high-copy vector [21, 29].

## Additional file

**Additional file 1.** Supplementary information.

## Abbreviations

CRISPR: clustered regularly interspaced short palindromic repeats
GD: gene drive
DSB: double strand break
HR: homologous recombination
HDR: homology directed repair
NHEJ: non-homologous end joining
sgRNA: single guide RNA fragment
NLS: nuclear localization sequence
NES: nuclear export signal
GPCR: G-protein coupled receptor
DIC: differential interference contrast
Pr: promoter
(t): terminator
S: synthetic complete media
