# Supplementary material for: Modulating CRISPR gene drive activity through nucleocytoplasmic localization of Cas9 in S. cerevisiae
Source: Fungal Biol Biotechnol. 2019 Feb 4;6:2. doi: 10.1186/s40694-019-0065-x (PMC6360766; doi:10.1186/s40694-019-0065-x)
Supplement: Supplementary file 1 — Additional file 1. Supplementary information. [file 40694_2019_65_MOESM1_ESM.pdf]

SUPPORTING INFORMATION

For

Modulating CRISPR gene drive activity through nucleocytoplasmic localization of Cas9 in *S. cerevisiae*

Megan E. Goeckel[1†], Erianna M. Basgall[1†], Isabel C. Lewis[1†], Samantha C. Goetting[1†], Yao Yan[1], Megan Halloran[1‡], and Gregory C. Finnigan[1*]

[1]Department of Biochemistry and Molecular Biophysics, 141 Chalmers Hall, Kansas State University, Manhattan, KS 66506 USA

[†]Authors contributed equally

[‡]Current address: Department of Psychology, 106-B Kastle Hall, University of Kentucky, Lexington, KY 40506 USA

*Correspondence to: Gregory C. Finnigan, Phone: (785) 532-6939; FAX; (785) 532-7278; E-mail: gfinnigan@ksu.edu

Figure S1. DNA sequences and maps of engineered CRISPR gene drive cassettes, target strains, and modified alleles within the yeast genome from strains within this study. Start and Stop codons are highlighted with a red background for clarity. Coding sequences are in bold type. Approximately 1,000 bp of flanking sequence (native yeast genome) is presented for each manipulated locus.

GFY-3206:

prHIS3::***(u1)***::*prCDC12*::***mCherry***::***NLS***^{SV40}::*SHS1(t)*::*prCCW12*::***SpHIS5***::*MX(t)*::***(u1)***::*HIS3(t)*

(u2): 20 bp target and 3 bp PAM sequence

(992 bp 5′ UTR to *HIS3* shown)

GGGTCAGTTATTTCATCCAGATATAACCCGAGAGGAAACTTCTTAGCGTCTGTTTTCGTACCATAAGGCAGTTCATG
AGGTATATTTTCGTTATTGAAGCCCAGCTCGTGAATGCTTAATGCTGCTGAACTGGTGTCCATGTCGCCTAGGTACG
CAATCTCCACAGGCTGCAAAGGTTTTGTCTCAAGAGCAATGTTATTGTGCACCCCGTAATTGGTCAACAAGTTTAAT
CTGTGCTTGTCCACCAGCTCTGTCGTAACCTTCAGTTCATCGACTATCTGAAGAAATTTACTAGGAATAGTGCCATG
GTACAGCAACCGAGAATGGCAATTTCTACTCGGGTTCAGCAACGCTGCATAAACGCTGTTGGTGCCGTAGACATATT
CGAAGATAGGATTATCATTCATAAGTTTCAGAGCAATGTCCTTATTCTGGAACTTGGATTTATGGCTCTTTTGGTTT
AATTTCGCCTGATTCTTGATCTCCTTTAGCTTCTCGACGTGGGCCTTTTTCTTGCCATATGGATCCGCTGCACGGTC
CTGTTCCCTAGCATGTACGTGAGCGTATTTCCTTTTAAACCACGACGCTTTGTCTTCATTCAACGTTTCCCATTGTT
TTTTTCTACTATTGCTTTGCTGTGGGAAAAACTTATCGAAAGATGACGACTTTTTCTTAATTCTCGTTTTAAGAGCT
TGGTGAGCGCTAGGAGTCACTGCCAGGTATCGTTTGAACACGGCATTAGTCAGGGAAGTCATAACACAGTCCTTTCC
CGCAATTTTCTTTTTCTATTACTCTTGGCCTCCTCTAGTACACTCTATATTTTTTTATGCCTCGGTAATGATTTTCA
TTTTTTTTTTTCCACCTAGCGGATGACTCTTTTTTTTTCTTAGCGATTGGCATTATCACATAATGAATTATACATTA
TATAAAGTAATGTGATTTCTTCGAAGAATATACTAAAAAATGAGCAGGCAAGATAAACGAAGGCAAAG**ATGACGGTG**
GACTTCGGCTACGTAGGGCGATTGGGGCAGCGCCCTGTTTTTCATTAATGTAGTCAGCAATGTCAAGATTCAACGCC
AAGTCTGGTTCAGCAAGTGACATTCTGCAAGCTCTTTGAATCTTCCTCAAAAGAGGATTGCCCAAGGCTTGAGGTTT
CCTGACGGGCAACTCAGACAAATATATGCTATGTGAGTGCGGATGGGACATGATGCAGTATCACGATTAGCAATTCA
GCTATGAGTTATGTTGCTCTTTGTTTTGTTTATGGAAATTGTCCTATGGTAAGTCTCTTTTTTTTGCAATCGTGATT
ACAGAAAAAAAACAGGGCGCTGGAAAAGTGAAGAATCCGAAATTTTTTTCGAAATCACCATTGTTTGTTTTGAGTAG
ATCAAAGTCTTGAAAGGTGCAGCAAGATATAGGATCTTGACCTGAAGAGTATTGATAACGAACTACATCACATATTG
TATCAAATA**ATGGTGAGCAAGGGCGAGGAGGATAACATGGCCATCATCAAGGAGTTCATGCGCTTCAAGGTGCACAT**
GGAGGGCTCCGTGAACGGCCACGAGTTCGAGATCGAGGGCGAGGGCGAGGGCCGCCCCTACGAGGGCACCCAGACCG
CCAAGCTGAAGGTGACCAAGGGTGGCCCCCTGCCCTTCGCCTGGGACATCCTGTCCCCTCAGTTCATGTACGGCTCC
AAGGCCTACGTGAAGCACCCCGCCGACATCCCCGACTACTTGAAGCTGTCCTTCCCCGAGGGCTTCAAGTGGGAGCG
CGTGATGAACTTCGAGGACGGCGGCGTGGTGACCGTGACCCAGGACTCCTCCCTGCAGGACGGCGAGTTCATCTACA
AGGTGAAGCTGCGCGGCACCAACTTCCCCTCCGACGGCCCCGTAATGCAGAAGAAGACCATGGGCTGGGAGGCCTCC
TCCGAGCGGATGTACCCCGAGGACGGCGCCCTGAAGGGCGAGATCAAGCAGAGGCTGAAGCTGAAGGACGGCGGCCA
CTACGACGCTGAGGTCAAGACCACCTACAAGGCCAAGAAGCCCGTGCAGCTGCCCGGCGCCTACAACGTCAACATCA
AGTTGGACATCACCTCCCACAACGAGGACTACACCATCGTGGAACAGTACGAACGCGCCGAGGGCCGCCACTCCACC
GGCGGCATGGACGAGCTGTACAAGTCTAGGGCAGACCCAAAGAAAAAGAGGAAAGTATAAGTTGTATCTGTACAAAA
TCCAAAGCTGAGCAAATAAATAAATAAATAAATGTATAAGTTACCGAACGGGGGTATTTTTACTTTTGATCAAAAAT
TTATGTACCAACTACAAAGTTTCCTCAGCACAGCCTTCAAGAAGGGAACACACATACAAACAGTGTCAAATAATTGT
AGGGATAAATTTAAATATGGCATAAACTAAATAAGTAGAGCATGAAAAAACTGCAAAATCCAAAAAGTAAAAACGAA
GGTCAGAAAGTAAAGCAAAAGAAAATTAATAAAGCAATACTAAATCTATCATGATTTCCCGTAACTTCCATTAAAGC
TGTAACCAGATTTACTCCTACTGTTTGAGCCTCTAACGCCTAATGGATTTTTAGAGAAGCTCAACCTGATACCTCCT
TTGTTGTTGAGGGAAGGGCGGGGGTGAGGTAGTTGACTACCATATAATTCTGCCAATGCTCTAGTGGCAAAGCTAAC
ATCCTCACAAAGCAAAATAAAAGAAACTTAATACGTTATGCCGTAATGAAGGGCTACCAAAAACGATAATCTCAACT
GTAAACAGGTACAATGCGGACCCTTTTGCCACAAAACATACATCATTCATTGCCGGAAAAAGAAAGAAGTGAAGACA

GCAGTGCAGCCAGCCATGTTGCGCCAATCTAATTATAGATGCTGGTGCCCTGAGGATGTATCTGGAGCCAGCCATGG
CATCATGCGCTACCGCCGGATGTAAAATCCGACACGCAAAAGAAAACCTTCGAGGTTGCGCACTTCGCCCACCCATG
AACCACACGGTTAGTCCAAAAGGGGCAGTTCAGATTCCAGATGCGGGAATTAGCTTGCTGCCACCCTCACCTCACTA
ACGCTGCGGTGTGCGGATACTTCATGCTATTTATAGACGCGCGTGTCGGAATCAGCACGCGCAAGAACCAAATGGGA
AAATCGGAATGGGTCCAGAACTGCTTTGAGTGCTGGCTATTGGCGTCTGATTTCCGTTTTGGGAATCCTTTGCCGCG
CGCCCCTCTCAAAACTCCGCACAAGTCCCAGAAAGCGGGAAAGAAATAAAACGCCACCAAAAAAAAAAAAAATAAAAG
CCAATCCTCGAAGCGTGGGTGGTAGGCCCTGGATTATCCCGTACAAGTATTTCTCAGGAGTAAAAAAACCGTTTGTT
TTGGAATTCCCCATTTCGCGGCCACCTACGCCGCTATCTTTGCAACAACTATCTGCGATAACTCAGCAAATTTTGCA
TATTCGTGTTGCAGTATTGCGATAATGGGAGTCTTACTTCCAACATAACGGCAGAAAGAAATGTGAGAAAATTTTGC
ATCCTTTGCCTCCGTTCAAGTATATAAAGTCGGCATGCTTGATAATCTTTCTTTCCATCCTACATTGTTCTAATTAT
TCTTATTCTCCTTTATTCTTTCCTAACATACCAAGAAATTAATCTTCTGTCATTCGCTTAAACACTATATCAATA**AT**
GAGGAGGGCTTTGTAGAAAGAAATACGAACGAAACGAAAATCAGCGTTGCCATCGCTTTGGACAAAGCTCCCTTAC
CTGAAGAGTCGAATTTTATTGATGAACTTATAACTTCCAAGCATACAAACCAAAAGGGAGAACAAGTAATCCAAGTA
GACACGGGAATTGGATTCTTGGATCACATGTATCATGCACTGGCTAAACATGCAGGCTGGAGCTTACGACTTTACTC
AAGAGGTGATTTAATCATCGATGATCATCACACTGCAGAAGATACTGCTATTGCACTTGGTATTGCATTCAAGCAGG
CTATGAGTAACTTTGCCGGCGTTAAAAGATTTGGACATGCTTATTGTCCACTTGACGAAGCTCTTTCTAGAAGCGTA
GTTGACTTGTCGGGACGGCCCTATGCTGTTATCGATTTGGGATTAAAGCGTGAAAAGGTTGGGGAATTGTCCTGTGA
AATGATCCCTCACTTACTATATTCCTTTTCGGTAGCAGCTGGAATTACTTTGCATGTTACCTGCTTATATGGTAGTA
ATGACCATCATCGTGCTGAAAGCGCTTTTAAATCTCTGGCTGTTGCCATGCGCGCGGCTACTAGTCTTACTGGAAGT
TCTGAAGTCCCAAGCACGAAGGGAGTGTTGTAAAGAGTACTGACAATAAAAAGATTCTTGTTTTCAAGAACTTGTCA
TTTGTATAGTTTTTTTATATTGTAGTTGTTCTATTTTAATCAAATGTTAGCGTGATTTATATTTTTTTTCGCCTCGA
CATCATCTGCCCAGATGCGAAGTTAAGTGCGCAGAAAGTAATATCATGCGTCAATCGTATGTGAATGCTGGTCGCTA
TACTGCTGTCGATTCGATACTAACGCCGCCATCCAGT**ATGACGGTGGACTTCGGCTACGTAGGGCGATT**TGACACCG
ATTATTTAAAGCTGCAGCATACGATATATATACATGTGTATATATGTATACCTATGAATGTCAGTAAGTATGTATAC
GAACAGTATGATACTGAAGATGACAAGGTAATGCATCATTCTATACGTGTCATTCTGAACGAGGCGCGCTTTCCTTT
TTTCTTTTTGCTTTTTCTTTTTTTTTCTCTTGAACTCGAGAAAAAAAAATATAAAAGAGATGGAGGAACGGGAAAAAG
TTAGTTGTGGTGATAGGTGGCAAGTGGTATTCCGTAAGAACAACAAGAAAAGCATTTCATATTATGGCTGAACTGAG
CGAACAAGTGCAAAATTTAAGCATCAACGACAACAACGAGAATGGTTATGTTCCTCCTCACTTAAGAGGAAAACCAA
GAAGTGCCAGAAATAACAGTAGCAACTACAATAACAACAACGGCGGCTACAACGGTGGCCGTGGCGGTGGCAGCTTC
TTTAGCAACAACCGTCGTGGTGGTTACGGCAACGGTGGTTTCTTCGGTGGAAACAACGGTGGCAGCAGATCTAACGG
CCGTTCTGGTGGTAGATGGATCGATGGCAAACATGTCCCAGCTCCAAGAAACGAAAAGGCCGAGATCGCCATATTTG
GTGTCCCCGAGGATCCAAATTTCCAATCTTCTGGTATTAACTTCGATAACTACGATGATATTCCAGTGGACGCCTCT
GGTAAGGATGTTCCTGAACCAATCACAGAATTTACCTCACCTCCATTGGACGGATTGTTATTGGAAAACATCAAATT
GGCCCGTTTCACCAAGCCAACACCTGTGCAAAAATACTCCGTCCCTATCGTTGCCAACGGCAGAGATTTGATGGCCT
GTGCGCAGACCGGTTCTGGTAAGACTGGTGGGTTTTTATTCCCAGTGTTGTCCGAATCATTTAAGACTGGACCATCT
CCTCAACCAGAGTCTCAAGGCTCCTTTTACCAAAGAAAGGCCTACCCAACTGCTGTC

(989 bp 3′ UTR to *HIS3* shown)

GFY-3207:

prHIS3::***(u1)***::*prCDC12*::***mCherry***::*SHS1(t)*::*prCCW12*::***SpHIS5***::*MX(t)*::***(u1)***::*HIS3(t)*

***(u1)*: *20 bp target* and *3 bp PAM* sequence**

(992 bp 5′ UTR to *HIS3* shown)

GGGTCAGTTATTTCATCCAGATATAACCCGAGAGGAAACTTCTTAGCGTCTGTTTTCGTACCATAAGGCAGTTCATG
AGGTATATTTTCGTTATTGAAGCCCAGCTCGTGAATGCTTAATGCTGCTGAACTGGTGTCCATGTCGCCTAGGTACG
CAATCTCCACAGGCTGCAAAGGTTTTGTCTCAAGAGCAATGTTATTGTGCACCCCGTAATTGGTCAACAAGTTTAAT
CTGTGCTTGTCCACCAGCTCTGTCGTAACCTTCAGTTCATCGACTATCTGAAGAAATTTACTAGGAATAGTGCCATG
GTACAGCAACCGAGAATGGCAATTTCTACTCGGGTTCAGCAACGCTGCATAAACGCTGTTGGTGCCGTAGACATATT
CGAAGATAGGATTATCATTCATAAGTTTCAGAGCAATGTCCTTATTCTGGAACTTGGATTTATGGCTCTTTTGGTTT
AATTTCGCCTGATTCTTGATCTCCTTTAGCTTCTCGACGTGGGCCTTTTTCTTGCCATATGGATCCGCTGCACGGTC
CTGTTCCCTAGCATGTACGTGAGCGTATTTCCTTTTAAACCACGACGCTTTGTCTTCATTCAACGTTTCCCATTGTT

TTTTTCTACTATTGCTTTGCTGTGGGAAAAACTTATCGAAAGATGACGACTTTTTCTTAATTCTCGTTTTAAGAGCT
TGGTGAGCGCTAGGAGTCACTGCCAGGTATCGTTTGAACACGGCATTAGTCAGGGAAGTCATAACACAGTCCTTTCC
CGCAATTTTCTTTTTCTATTACTCTTGGCCTCCTCTAGTACACTCTATATTTTTTTATGCCTCGGTAATGATTTTCA
TTTTTTTTTTTCCACCTAGCGGATGACTCTTTTTTTTTCTTAGCGATTGGCATTATCACATAATGAATTATACATTA
TATAAAGTAATGTGATTTCTTCGAAGAATATACTAAAAAATGAGCAGGCAAGATAAACGAAGGCAAAGATGACGGTG
GACTTCGGCTACGTAGGGCGATTGGGGCAGCGCCCTGTTTTTCATTAATGTAGTCAGCAATGTCAAGATTCAACGCC
AAGTCTGGTTCAGCAAGTGACATTCTGCAAGCTCTTTGAATCTTCCTCAAAAGAGGATTGCCCAAGGCTTGAGGTTT
CCTGACGGGCAACTCAGACAAATATATGCTATGTGAGTGCGGATGGGACATGATGCAGTATCACGATTAGCAATTCA
GCTATGAGTTATGTTGCTCTTTGTTTTGTTTATGGAAATTGTCCTATGGTAAGTCTCTTTTTTTTGCAATCGTGATT
ACAGAAAAAAAACAGGGCGCTGGAAAAGTGAAGAATCCGAAATTTTTTTCGAAATCACCATTGTTTGTTTTGAGTAG
ATCAAAGTCTTGAAAGGTGCAGCAAGATATAGGATCTTGACCTGAAGAGTATTGATAACGAACTACATCACATATTG
TATCAAATAATGGTGAGCAAGGGCGAGGAGGATAACATGGCCATCATCAAGGAGTTCATGCGCTTCAAGGTGCACAT
GGAGGGCTCCGTGAACGGCCACGAGTTCGAGATCGAGGGCGAGGGCGAGGGCCGCCCCTACGAGGGCACCCAGACCG
CCAAGCTGAAGGTGACCAAGGGTGGCCCCCTGCCCTTCGCCTGGGACATCCTGTCCCCTCAGTTCATGTACGGCTCC
AAGGCCTACGTGAAGCACCCCGCCGACATCCCCGACTACTTGAAGCTGTCCTTCCCCGAGGGCTTCAAGTGGGAGCG
CGTGATGAACTTCGAGGACGGCGGCGTGGTGACCGTGACCCAGGACTCCTCCCTGCAGGACGGCGAGTTCATCTACA
AGGTGAAGCTGCGCGGCACCAACTTCCCCTCCGACGGCCCCGTAATGCAGAAGAAGACCATGGGCTGGGAGGCCTCC
TCCGAGCGGATGTACCCCGAGGACGGCGCCCTGAAGGGCGAGATCAAGCAGAGGCTGAAGCTGAAGGACGGCGGCCA
CTACGACGCTGAGGTCAAGACCACCTACAAGGCCAAGAAGCCCGTGCAGCTGCCCGGCGCCTACAACGTCAACATCA
AGTTGGACATCACCTCCCACAACGAGGACTACACCATCGTGGAACAGTACGAACGCGCCGAGGGCCGCCACTCCACC
GGCGGCATGGACGAGCTGTACAAGTAAGTTGTATCTGTACAAAATCCAAAGCTGAGCAAATAAATAAATAAATAAAT
GTATAAGTTACCGAACGGGGGTATTTTTACTTTTGATCAAAAATTTATGTACCAACTACAAAGTTTCCTCAGCACAG
CCTTCAAGAAGGGAACACACATACAAACAGTGTCAAATAATTGTAGGGATAAATTTAAATATGGCATAAACTAAATA
AGTAGAGCATGAAAAAACTGCAAAATCCAAAAAGTAAAAACGAAGGTCAGAAAGTAAAGCAAAAGAAAATTAATAAA
GCAATACTAAATCTATCATGATTTCCCGTAACTTCCATTAAAGCTGTAACCAGATTTACTCCTACTGTTTGAGCCTC
TAACGCCTAATGGATTTTTAGAGAAGCTCAACCTGATACCTCCTTTGTTGTTGAGGGAAGGGCGGGGGTGAGGTAGT
TGACTACCATATAATTCTGCCAATGCTCTAGTGGCAAAGCTAACATCCTCACAAAGCAAAATAAAAGAAACTTAATA
CGTTATGCCGTAATGAAGGGCTACCAAAAACGATAATCTCAACTGTAAACAGGTACAATGCGGACCCTTTTGCCACA
AAACATACATCATTCATTGCCGGAAAAAGAAAGAAGTGAAGACAGCAGTGCAGCCAGCCATGTTGCGCCAATCTAAT
TATAGATGCTGGTGCCCTGAGGATGTATCTGGAGCCAGCCATGGCATCATGCGCTACCGCCGGATGTAAAATCCGAC
ACGCAAAAGAAAACCTTCGAGGTTGCGCACTTCGCCCACCCATGAACCACACGGTTAGTCCAAAAGGGGCAGTTCAG
ATTCCAGATGCGGGAATTAGCTTGCTGCCACCCTCACCTCACTAACGCTGCGGTGTGCGGATACTTCATGCTATTTA
TAGACGCGCGTGTCGGAATCAGCACGCGCAAGAACCAAATGGGAAAATCGGAATGGGTCCAGAACTGCTTTGAGTGC
TGGCTATTGGCGTCTGATTTCCGTTTTGGGAATCCTTTGCCGCGCGCCCCTCTCAAAACTCCGCACAAGTCCCAGAA
AGCGGGAAAGAAATAAAACGCCACCAAAAAAAAAAAAAATAAAAGCCAATCCTCGAAGCGTGGGTGGTAGGCCCTGGA
TTATCCCGTACAAGTATTTCTCAGGAGTAAAAAAACCGTTTGTTTTGGAATTCCCCATTTCGCGGCCACCTACGCCG
CTATCTTTGCAACAACTATCTGCGATAACTCAGCAAATTTTGCATATTCGTGTTGCAGTATTGCGATAATGGGAGTC
TTACTTCCAACATAACGGCAGAAAGAAATGTGAGAAAATTTTGCATCCTTTGCCTCCGTTCAAGTATATAAAGTCGG
CATGCTTGATAATCTTTCTTTCCATCCTACATTGTTCTAATTATTCTTATTCTCCTTTATTCTTTCCTAACATACCA
AGAAATTAATCTTCTGTCATTCGCTTAAACACTATATCAATAATGAGGAGGGCTTTTGTAGAAAGAAATACGAACGA
AACGAAAATCAGCGTTGCCATCGCTTTGGACAAAGCTCCCTTACCTGAAGAGTCGAATTTTATTGATGAACTTATAA
CTTCCAAGCATACAAACCAAAAGGGAGAACAAGTAATCCAAGTAGACACGGGAATTGGATTCTTGGATCACATGTAT
CATGCACTGGCTAAACATGCAGGCTGGAGCTTACGACTTTACTCAAGAGGTGATTTAATCATCGATGATCATCACAC
TGCAGAAGATACTGCTATTGCACTTGGTATTGCATTCAAGCAGGCTATGAGTAACTTTGCCGGCGTTAAAAGATTTG
GACATGCTTATTGTCCACTTGACGAAGCTCTTTCTAGAAGCGTAGTTGACTTGTCGGGACGGCCCTATGCTGTTATC
GATTTGGGATTAAAGCGTGAAAAGGTTGGGGAATTGTCCTGTGAAATGATCCCTCACTTACTATATTCCTTTTCGGT
AGCAGCTGGAATTACTTTGCATGTTACCTGCTTATATGGTAGTAATGACCATCATCGTGCTGAAAGCGCTTTTAAAT
CTCTGGCTGTTGCCATGCGCGCGGCTACTAGTCTTACTGGAAGTTCTGAAGTCCCAAGCACGAAGGGAGTGTTGTAA
AGAGTACTGACAATAAAAAGATTCTTGTTTTCAAGAACTTGTCATTTGTATAGTTTTTTTATATTGTAGTTGTTCTA
TTTTAATCAAATGTTAGCGTGATTTATATTTTTTTTCGCCTCGACATCATCTGCCCAGATGCGAAGTTAAGTGCGCA
GAAAGTAATATCATGCGTCAATCGTATGTGAATGCTGGTCGCTATACTGCTGTCGATTCGATACTAACGCCGCCATC
CAGTATGACGGTGGACTTCGGCTACGTAGGGCGATTTGACACCGATTATTTAAAGCTGCAGCATACGATATATATAC
ATGTGTATATATGTATACCTATGAATGTCAGTAAGTATGTATACGAACAGTATGATACTGAAGATGACAAGGTAATG
CATCATTCTATACGTGTCATTCTGAACGAGGCGCGCTTTCCTTTTTTCTTTTTGCTTTTTCTTTTTTTTTTCTCTTGA
ACTCGAGAAAAAAAATATAAAAGAGATGGAGGAACGGGAAAAAGTTAGTTGTGGTGATAGGTGGCAAGTGGTATTCC
GTAAGAACAACAAGAAAAGCATTTCATATTATGGCTGAACTGAGCGAACAAGTGCAAAATTTAAGCATCAACGACAA
CAACGAGAATGGTTATGTTCCTCCTCACTTAAGAGGAAAACCAAGAAGTGCCAGAAATAACAGTAGCAACTACAATA

ACAACAACGGCGGCTACAACGGTGGCCGTGGCGGTGGCAGCTTCTTTAGCAACAACCGTCGTGGTGGTTACGGCAAC
GGTGGTTTCTTCGGTGGAAACAACGGTGGCAGCAGATCTAACGGCCGTTCTGGTGGTAGATGGATCGATGGCAAACA
TGTCCCAGCTCCAAGAAACGAAAAGGCCGAGATCGCCATATTTGGTGTCCCCGAGGATCCAAATTTCCAATCTTCTG
GTATTAACTTCGATAACTACGATGATATTCCAGTGGACGCCTCTGGTAAGGATGTTCCTGAACCAATCACAGAATTT
ACCTCACCTCCATTGGACGGATTGTTATTGGAAAACATCAAATTGGCCCGTTTCACCAAGCCAACACCTGTGCAAAA
ATACTCCGTCCCTATCGTTGCCAACGGCAGAGATTTGATGGCCTGTGCGCAGACCGGTTCTGGTAAGACTGGTGGGT
TTTTATTCCCAGTGTTGTCCGAATCATTTAAGACTGGACCATCTCCTCAACCAGAGTCTCAAGGCTCCTTTTACCAA
AGAAAGGCCTACCCAACTGCTGTC

(989 bp 3′ UTR to *HIS3* shown)

- -

GFY-2756:

prHIS3::***(u2)***::*prGAL1/10*::***SpCas9***::***eGFP***::***NLS***^{SV40}::*ADH1(t)*::*prMX*::***Kan***^{R}::*MX(t)*::***(u2)***::*HIS3(t)*

(u2) 20 bp target and 3 bp PAM sequence

(992 bp 5′ UTR to *HIS3* shown)
GGGTCAGTTATTTCATCCAGATATAACCCGAGAGGAAACTTCTTAGCGTCTGTTTTCGTACCATAAGGCAGTTCATG
AGGTATATTTTCGTTATTGAAGCCCAGCTCGTGAATGCTTAATGCTGCTGAACTGGTGTCCATGTCGCCTAGGTACG
CAATCTCCACAGGCTGCAAAGGTTTTGTCTCAAGAGCAATGTTATTGTGCACCCCGTAATTGGTCAACAAGTTTAAT
CTGTGCTTGTCCACCAGCTCTGTCGTAACCTTCAGTTCATCGACTATCTGAAGAAATTTACTAGGAATAGTGCCATG
GTACAGCAACCGAGAATGGCAATTTCTACTCGGGTTCAGCAACGCTGCATAAACGCTGTTGGTGCCGTAGACATATT
CGAAGATAGGATTATCATTCATAAGTTTCAGAGCAATGTCCTTATTCTGGAACTTGGATTTATGGCTCTTTTGGTTT
AATTTCGCCTGATTCTTGATCTCCTTTAGCTTCTCGACGTGGGCCTTTTTCTTGCCATATGGATCCGCTGCACGGTC
CTGTTCCCTAGCATGTACGTGAGCGTATTTCCTTTTAAACCACGACGCTTTGTCTTCATTCAACGTTTCCCATTGTT
TTTTTCTACTATTGCTTTGCTGTGGGAAAAACTTATCGAAAGATGACGACTTTTTCTTAATTCTCGTTTTAAGAGCT
TGGTGAGCGCTAGGAGTCACTGCCAGGTATCGTTTGAACACGGCATTAGTCAGGGAAGTCATAACACAGTCCTTTCC
CGCAATTTTCTTTTTCTATTACTCTTGGCCTCCTCTAGTACACTCTATATTTTTTTATGCCTCGGTAATGATTTTCA
TTTTTTTTTTTCCACCTAGCGGATGACTCTTTTTTTTTCTTAGCGATTGGCATTATCACATAATGAATTATACATTA
TATAAAGTAATGTGATTTCTTCGAAGAATATACTAAAAAATGAGCAGGCAAGATAAACGAAGGCAAAG**GCTGTTCGT**
GTGCGCGTCCTGGGGACAGGTTATCAGCAACAACACAGTCATATCCATTCTCAATTAGCTCTACCACAGTGTGTGAA
CCAATGTATCCAGCACCACCTGTAACCAAAACAATTTTAGAAGTACTTTCACTTTGTAACTGAGCTGTCATTTATAT
TGAATTTTCAAAAATTCTTACTTTTTTTTTGGATGGACGCAAAGAAGTTTAATAATCATATTACATGGCATTACCAC
CATATACATATCCATATACATATCCATATCTAATCTTACTTATATGTTGTGGAAATGTAAAGAGCCCCATTATCTTA
GCCTAAAAAAACCTTCTCTTTGGAACTTTCAGTAATACGCTTAACTGCTCATTGCTATATTGAAGTACGGATTAGAA
GCCGCCGAGCGGGTGACAGCCCTCCGAAGGAAGACTCTCCTCCGTGCGTCCTCGTCTTCACCGGTCGCGTTCCTGAA
ACGCAGATGTGCCTCGCGCCGCACTGCTCCGAACAATAAAGATTCTACAATACTAGCTTTTATGGTTATGAAGAGGA
AAAATTGGCAGTAACCTGGCCCCACAAACCTTCAAATGAACGAATCAAATTAACAACCATAGGATGATAATGCGATT
AGTTTTTTAGCCTTATTTCTGGGGTAATTAATCAGCGAAGCGATGATTTTTGATCTATTAACAGATATATAAATGCA
AAAACTGCATAACCACTTTAACTAATACTTTCAACATTTTCGGTTTGTATTACTTCTTATTCAAATGTAATAAAAGT
ATCAACAAAAAATTGTTAATATACCTCTATACTTTAACGTCAAGGAGAAAAAACTATA**ATGGATAAGAAATACTCTA**
TCGGTTTGGATATTGGTACAAATTCAGTTGGTTGGGCAGTTATTACTGATGAATACAAGGTTCCATCTAAAAAGTTT
AAAGTTTTGGGTAACACTGATAGACATTCTATTAAGAAAAATTTGATTGGTGCTTTGTTATTTGATTCTGGTGAAAC
TGCTGAAGCAACAAGATTGAAAAGAACTGCAAGAAGAAGATACACAAGAAGAAAGAATAGAATCTGTTATTTGCAAG
AAATTTTCTCTAACGAAATGGCTAAGGTTGATGATTCTTTCTTTCATAGATTGGAAGAATCATTTTTAGTTGAAGAA
GATAAGAAACATGAAAGACATCCAATCTTCGGTAACATCGTTGATGAAGTTGCTTACCATGAAAAGTACCCAACAAT
CTATCATTTGAGAAAGAAATTGGTTGATTCAACTGATAAGGCAGATTTGAGATTGATATATTTGGCTTTAGCACATA
TGATCAAGTTTAGAGGTCATTTCTTGATCGAGGGTGACTTGAATCCAGATAATTCTGATGTTGATAAGTTGTTTATT
CAATTAGTTCAAACATATAATCAATTGTTTGAAGAAAATCCAATTAATGCTTCTGGTGTTGATGCTAAGGCAATCTT
GTCAGCAAGATTGTCTAAGTCAAGAAGATTGGAAAATTTGATCGCTCAATTACCAGGTGAAAAGAAAAATGGTTTGT
TCGGTAATTTGATCGCATTGTCTTTGGGTTTGACACCAAACTTCAAGTCAAACTTCGATTTGGCTGAAGATGCAAAG
TTGCAATTGTCTAAGGATACTTACGATGATGATTTGGATAATTTGTTGGCTCAAATTGGTGACCAATATGCAGATTT
GTTTTTGGCTGCTAAAAATTTGTCTGATGCTATCTTGTTGTCAGATATCTTGAGAGTTAACACTGAAATCACAAAGG

CTCCATTGTCTGCATCAATGATCAAGAGATACGATGAACATCATCAAGATTTGACTTTGTTGAAGGCATTGGTTAGA
CAACAATTACCAGAAAAGTACAAGGAAATTTTCTTTGATCAATCTAAAAATGGTTATGCTGGTTACATTGATGGTGG
TGCATCTCAAGAAGAATTCTACAAGTTTATTAAGCCAATCTTGGAAAAGATGGATGGTACAGAAGAATTGTTAGTTA
AATTGAACAGAGAAGATTTGTTAAGAAAACAAAGAACTTTCGATAACGGTTCTATCCCACATCAAATCCATTTGGGT
GAATTACATGCTATCTTGAGAAGACAAGAAGATTTCTACCCATTTTTAAAGGATAACAGAGAAAAGATTGAAAAGAT
TTTGACTTTTAGAATTCCATATTACGTTGGTCCATTAGCTCGTGGTAATTCTAGATTTGCATGGATGACTAGAAAGT
CAGAAGAAACTATCACACCATGGAATTTTGAAGAAGTTGTTGATAAAGGTGCTTCTGCACAATCTTTTATTGAAAGA
ATGACAAACTTCGATAAAAATTTGCCAAACGAAAAGGTTTTGCCAAAGCATTCATTGTTATATGAATACTTTACTGT
TTACAATGAATTGACAAAAGTTAAATATGTTACTGAGGGTATGAGAAAACCAGCATTTTTGTCTGGTGAACAAAAGA
AAGCAATCGTTGATTTGTTGTTTAAAACTAACAGAAAGGTTACAGTTAAACAATTGAAAGAAGATTACTTTAAGAAA
ATTGAATGTTTTGATTCTGTTGAAATTTCAGGTGTTGAAGATAGATTCAATGCTTCATTAGGTACTTACCATGATTT
GTTGAAGATTATTAAGGATAAAGATTTCTTGGATAATGAAGAAAATGAAGATATTTTAGAAGATATTGTTTTAACTT
TGACATTATTTGAAGATAGAGAAATGATCGAAGAAAGATTGAAGACATACGCTCATTTGTTCGATGATAAAGTTATG
AAGCAATTGAAGAGAAGAAGATACACTGGTTGGGGTAGATTGTCTAGAAAGTTGATTAATGGTATCAGAGATAAGCA
ATCTGGTAAAACAATCTTGGATTTCTTGAAGTCAGATGGTTTCGCAAACAGAAACTTCATGCAATTGATTCATGATG
ATTCATTGACTTTTAAAGAAGATATCCAAAAAGCTCAAGTTTCTGGTCAGGGTGACTCATTGCATGAACATATTGCT
AATTTGGCAGGTTCTCCAGCTATTAAGAAAGGTATCTTGCAAACAGTTAAGGTTGTTGATGAATTAGTTAAAGTTAT
GGGTAGACATAAGCCAGAAAACATCGTTATCGAAATGGCTAGAGAAAACCAAACTACACAAAAGGGTCAAAAGAATT
CAAGAGAAAGAATGAAGAGAATCGAAGAAGGTATTAAAGAATTGGGTTCTCAAATCTTGAAGGAACATCCAGTTGAA
AACACTCAATTGCAAAACGAAAAGTTGTACTTATACTACTTACAAAACGGTAGAGATATGTACGTTGATCAAGAATT
AGATATCAACAGATTGTCAGATTACGATGTTGATCATATCGTTCCACAATCATTTTTGAAGGATGATTCAATCGATA
ATAAGGTTTTGACAAGATCTGATAAGAACCGTGGTAAATCTGATAATGTTCCATCAGAAGAAGTTGTTAAGAAAATG
AAGAACTACTGGAGACAATTGTTAAATGCTAAGTTGATCACTCAAAGAAAGTTCGATAATTTGACAAAAGCTGAAAG
AGGTGGTTTGTCAGAATTAGATAAAGCAGGTTTTATTAAGAGACAATTAGTTGAAACTAGACAAATCACAAAGCATG
TTGCACAAATCTTGGATTCTAGAATGAACACTAAATATGATGAAAATGATAAATTAATTAGAGAAGTTAAAGTTATT
ACATTAAAATCTAAATTGGTTTCAGATTTTAGAAAAGATTTTCAATTCTACAAAGTTAGAGAAATTAATAACTATCA
TCATGCTCATGATGCATACTTGAATGCTGTTGTTGGTACTGCATTGATTAAGAAATACCCAAAGTTGGAATCTGAAT
TCGTTTACGGTGACTACAAGGTTTACGATGTTAGAAAGATGATCGCTAAGTCAGAACAAGAAATCGGTAAAGCTACA
GCAAAGTATTTCTTTTATTCTAACATCATGAATTTCTTTAAAACTGAAATTACATTAGCTAACGGTGAAATCAGAAA
AAGACCATTGATCGAAACTAATGGTGAAACAGGTGAAATTGTTTGGGATAAAGGTAGAGATTTCGCAACTGTTAGAA
AGGTTTTGTCAATGCCACAAGTTAACATCGTTAAGAAAACTGAAGTTCAAACAGGTGGTTTTTCTAAGGAATCAATC
TTGCCAAAGAGAAACTCTGATAAGTTGATTGCTAGAAAGAAAGATTGGGATCCAAAGAAATATGGTGGTTTTGATTC
TCCAACTGTTGCTTACTCAGTTTTAGTTGTTGCAAAGGTTGAAAAGGGTAAATCTAAGAAATTGAAATCAGTTAAAG
AATTGTTAGGTATCACAATCATGGAAAGATCTTCATTCGAAAAGAATCCAATCGATTTCTTGGAAGCAAAGGGTTAC
AAGGAAGTTAAGAAAGATTTGATTATTAAGTTGCCAAAGTACTCTTTGTTCGAATTAGAAAACGGTAGAAAAAGAAT
GTTAGCTTCAGCTGGTGAATTGCAAAAGGGTAATGAATTGGCTTTGCCATCTAAGTACGTTAATTTCTTGTATTTGG
CATCTCATTACGAAAAGTTGAAGGGTTCACCAGAAGATAATGAACAAAAACAATTGTTCGTTGAACAACATAAGCAT
TATTTGGATGAAATTATTGAACAAATTTCTGAATTTTCAAAAAGAGTTATTTTGGCTGATGCAAATTTGGATAAGGT
TTTGTCTGCTTACAATAAGCATAGAGATAAGCCAATCAGAGAACAAGCAGAAAACATCATCCATTTGTTTACTTTGA
CAAATTTGGGTGCTCCAGCTGCTTTTAAATACTTCGATACTACAATCGATAGAAAAAGATACACTTCTACAAAGGAA
GTTTTGGATGCAACATTGATCCATCAATCAATCACTGGTTTGTATGAAACAAGAATTGATTTGTCTCAATTGGGTGG
TGACGGTCGACGGATCCCCGGGTTAATTAACAGTAAAGGAGAAGAACTTTTCACTGGAGTTGTCCCAATTCTTGTTG
AATTAGATGGTGATGTTAATGGGCACAAATTTTCTGTCAGTGGGGAGGGTGAAGGTGATGCAACATACGGAAAACTT
ACCCTTAAATTTATTTGCACTACTGGAAAACTACCTGTTCCATGGCCAACACTTGTCACTACTTTGACTTATGGTGT
TCAATGCTTTTCAAGATACCCAGATCATATGAAACAACATGACTTTTTCAAGAGTGCCATGCCCGAAGGTTATGTAC
AGGAAAGAACTATATTTTTCAAAGATGACGGGAACTACAAGACACGTGCTGAAGTCAAGTTTGAAGGTGATACCCTT
GTTAATAGAATCGAGTTAAAAGGTATTGATTTTAAAGAAGATGGAAACATTCTTGGACACAAATTGGAATACAACTA
TAACTCACACAATGTATACATCATGGCAGACAAACAAAAGAATGGAATCAAAGTTAACTTCAAAATTAGACACAACA
TTGAAGATGGAAGCGTTCAACTAGCAGACCATTATCAACAAAATACTCCAATTGGCGATGGCCCTGTCCTTTTACCA
GACAACCATTACCTGTCCACACAATCTGCCCTTTCGAAAGATCCCAACGAAAAGAGAGACCACATGGTCCTTCTTGA
GTTTGTAACAGCTGCTGGGATTACATTGGGCATGGATGAACTATACAAATCCAGAGCCGACCCCAAGAAAAAAAGAA
AAGTGTAGGGCGCGCCACTTCTAAATAAGCGAATTTCTTATGATTTATGATTTTTATTATTAAATAAGTTATAAAAA
AAATAAGTGTATACAAATTTTAAAGTGACTCTTAGGTTTTAAAACGAAAATTCTTATTCTTGAGTAACTCTTTCCTG
TAGGTCAGGTTGCTTTCTCAGGTATAGTATGAGGTCGCTCTTATTGACCACACCTCTACCGGCAGATCCGCTAGGGA
TAACAGGGTAATATAGATCTGTTTAGCTTGCCTCGTCCCCGCCGGGTCACCCGGCCAGCGACATGGAGGCCCAGAAT
ACCCTCCTTGACAGTCTTGACGTGCGCAGCTCAGGGGCATGATGTGACTGTCGCCCGTACATTTAGCCCATACATCC
CCATGTATAATCATTTGCATCCATACATTTTGATGGCCGCACGGCGCGAAGCAAAAATTACGGCTCCTCGCTGCAGA

CCTGCGAGCAGGGAAACGCTCCCCTCACAGACGCGTTGAATTGTCCCCACGCCGCGCCCCTGTAGAGAAATATAAAA
GGTTAGGATTTGCCACTGAGGTTCTTCTTTCATATACTTCCTTTTAAAATCTTGCTAGGATACAGTTCTCACATCAC
ATCCGAACATAAACAACC**ATGGGTAAGGAAAAGACTCACGTTTCGAGGCCGCGATTAAATTCCAACATGGATGCTGA**
TTTATATGGGTATAAATGGGCTCGCGATAATGTCGGGCAATCAGGTGCGACAATCTATCGATTGTATGGGAAGCCCG
ATGCGCCAGAGTTGTTTCTGAAACATGGCAAAGGTAGCGTTGCCAATGATGTTACAGATGAGATGGTCAGACTAAAC
TGGCTGACGGAATTTATGCCTCTTCCGACCATCAAGCATTTTATCCGTACTCCTGATGATGCATGGTTACTCACCAC
TGCGATCCCCGGCAAAACAGCATTCCAGGTATTAGAAGAATATCCTGATTCAGGTGAAAATATTGTTGATGCGCTGG
CAGTGTTCCTGCGCCGGTTGCATTCGATTCCTGTTTGTAATTGTCCTTTTAACAGCGATCGCGTATTTCGTCTCGCT
CAGGCGCAATCACGAATGAATAACGGTTTGGTTGATGCGAGTGATTTTGATGACGAGCGTAATGGCTGGCCTGTTGA
ACAAGTCTGGAAAGAAATGCATAAGCTTTTGCCATTCTCACCGGATTCAGTCGTCACTCATGGTGATTTCTCACTTG
ATAACCTTATTTTTGACGAGGGGAAATTAATAGGTTGTATTGATGTTGGACGAGTCGGAATCGCAGACCGATACCAG
GATCTTGCCATCCTATGGAACTGCCTCGGTGAGTTTTCTCCTTCATTACAGAAACGGCTTTTTCAAAAATATGGTAT
TGATAATCCTGATATGAATAAATTGCAGTTTCATTTGATGCTCGATGAGTTTTTCTAATCAGTACTGACAATAAAAA
GATTCTTGTTTTCAAGAACTTGTCATTTGTATAGTTTTTTTATATTGTAGTTGTTCTATTTTAATCAAATGTTAGCG
TGATTTATATTTTTTTTCGCCTCGACATCATCTGCCCAGATGCGAAGTTAAGTGCGCAGAAAGTAATATCATGCGTC
AATCGTATGTGAATGCTGGTCGCTATACTGCTGTCGATTCGATACTAACGCCGCCATCCAGT**GCTGTTCGTGTGCGC**
GTCCTGGGTGACACCGATTATTTAAAGCTGCAGCATACGATATATATACATGTGTATATATGTATACCTATGAATGT
CAGTAAGTATGTATACGAACAGTATGATACTGAAGATGACAAGGTAATGCATCATTCTATACGTGTCATTCTGAACG
AGGCGCGCTTTCCTTTTTTCTTTTTGCTTTTTCTTTTTTTTTCTCTTGAACTCGAGAAAAAAAATATAAAAGAGATG
GAGGAACGGGAAAAAGTTAGTTGTGGTGATAGGTGGCAAGTGGTATTCCGTAAGAACAACAAGAAAAGCATTTCATA
TTATGGCTGAACTGAGCGAACAAGTGCAAAATTTAAGCATCAACGACAACAACGAGAATGGTTATGTTCCTCCTCAC
TTAAGAGGAAAACCAAGAAGTGCCAGAAATAACAGTAGCAACTACAATAACAACAACGGCGGCTACAACGGTGGCCG
TGGCGGTGGCAGCTTCTTTAGCAACAACCGTCGTGGTGGTTACGGCAACGGTGGTTTCTTCGGTGGAAACAACGGTG
GCAGCAGATCTAACGGCCGTTCTGGTGGTAGATGGATCGATGGCAAACATGTCCCAGCTCCAAGAAACGAAAAGGCC
GAGATCGCCATATTTGGTGTCCCCGAGGATCCAAATTTCCAATCTTCTGGTATTAACTTCGATAACTACGATGATAT
TCCAGTGGACGCCTCTGGTAAGGATGTTCCTGAACCAATCACAGAATTTACCTCACCTCCATTGGACGGATTGTTAT
TGGAAAACATCAAATTGGCCCGTTTCACCAAGCCAACACCTGTGCAAAAATACTCCGTCCCTATCGTTGCCAACGGC
AGAGATTTGATGGCCTGTGCGCAGACCGGTTCTGGTAAGACTGGTGGGTTTTTATTCCCAGTGTTGTCCGAATCATT
TAAGACTGGACCATCTCCTCAACCAGAGTCTCAAGGCTCCTTTTACCAAAGAAAGGCCTACCCAACTGCTGTCATTA
(989 bp 3′ UTR to *HIS3* shown)

\- -

GFY-3470:

prHIS3::***(u2)***::*prGAL1/10*::***SpCas9***::***eGFP***::***(NLS^{SV40})$_2$***::*CDC10(t)*::*prMX*::***CaURA3***::*MX(t)*::***(u2)***::*HIS3(t)*

(u2) 20 bp target and 3 bp PAM sequence

(992 bp 5′ UTR to *HIS3* shown)
GGGTCAGTTATTTCATCCAGATATAACCCGAGAGGAAACTTCTTAGCGTCTGTTTTCGTACCATAAGGCAGTTCATG
AGGTATATTTTCGTTATTGAAGCCCAGCTCGTGAATGCTTAATGCTGCTGAACTGGTGTCCATGTCGCCTAGGTACG
CAATCTCCACAGGCTGCAAAGGTTTTGTCTCAAGAGCAATGTTATTGTGCACCCCGTAATTGGTCAACAAGTTTAAT
CTGTGCTTGTCCACCAGCTCTGTCGTAACCTTCAGTTCATCGACTATCTGAAGAAATTTACTAGGAATAGTGCCATG
GTACAGCAACCGAGAATGGCAATTTCTACTCGGGTTCAGCAACGCTGCATAAACGCTGTTGGTGCCGTAGACATATT
CGAAGATAGGATTATCATTCATAAGTTTCAGAGCAATGTCCTTATTCTGGAACTTGGATTTATGGCTCTTTTGGTTT
AATTTCGCCTGATTCTTGATCTCCTTTAGCTTCTCGACGTGGGCCTTTTTCTTGCCATATGGATCCGCTGCACGGTC
CTGTTCCCTAGCATGTACGTGAGCGTATTTCCTTTTAAACCACGACGCTTTGTCTTCATTCAACGTTTCCCATTGTT
TTTTTCTACTATTGCTTTGCTGTGGGAAAAACTTATCGAAAGATGACGACTTTTTCTTAATTCTCGTTTTAAGAGCT
TGGTGAGCGCTAGGAGTCACTGCCAGGTATCGTTTGAACACGGCATTAGTCAGGGAAGTCATAACACAGTCCTTTCC
CGCAATTTTCTTTTTCTATTACTCTTGGCCTCCTCTAGTACACTCTATATTTTTTTATGCCTCGGTAATGATTTTCA
TTTTTTTTTTTCCACCTAGCGGATGACTCTTTTTTTTTCTTAGCGATTGGCATTATCACATAATGAATTATACATTA
TATAAAGTAATGTGATTTCTTCGAAGAATATACTAAAAAATGAGCAGGCAAGATAAACGAAGGCAAAG**GCTGTTCGT**
GTGCGCGTCCTGGGGACAGGTTATCAGCAACAACACAGTCATATCCATTCTCAATTAGCTCTACCACAGTGTGTGAA
CCAATGTATCCAGCACCACCTGTAACCAAAACAATTTTAGAAGTACTTTCACTTTGTAACTGAGCTGTCATTTATAT

TGAATTTTCAAAAATTCTTACTTTTTTTTTGGATGGACGCAAAGAAGTTTAATAATCATATTACATGGCATTACCAC
CATATACATATCCATATACATATCCATATCTAATCTTACTTATATGTTGTGGAAATGTAAAGAGCCCCATTATCTTA
GCCTAAAAAAACCTTCTCTTTGGAACTTTCAGTAATACGCTTAACTGCTCATTGCTATATTGAAGTACGGATTAGAA
GCCGCCGAGCGGGTGACAGCCCTCCGAAGGAAGACTCTCCTCCGTGCGTCCTCGTCTTCACCGGTCGCGTTCCTGAA
ACGCAGATGTGCCTCGCGCCGCACTGCTCCGAACAATAAAGATTCTACAATACTAGCTTTTATGGTTATGAAGAGGA
AAAATTGGCAGTAACCTGGCCCCACAAACCTTCAAATGAACGAATCAAATTAACAACCATAGGATGATAATGCGATT
AGTTTTTTAGCCTTATTTCTGGGGTAATTAATCAGCGAAGCGATGATTTTTGATCTATTAACAGATATATAAATGCA
AAAACTGCATAACCACTTTAACTAATACTTTCAACATTTTCGGTTTGTATTACTTCTTATTCAAATGTAATAAAAGT
ATCAACAAAAAATTGTTAATATACCTCTATACTTTAACGTCAAGGAGAAAAAACTATA**ATGGATAAGAAATACTCTA**
TCGGTTTGGATATTGGTACAAATTCAGTTGGTTGGGCAGTTATTACTGATGAATACAAGGTTCCATCTAAAAAGTTT
AAAGTTTTGGGTAACACTGATAGACATTCTATTAAGAAAAATTTGATTGGTGCTTTGTTATTTGATTCTGGTGAAAC
TGCTGAAGCAACAAGATTGAAAAGAACTGCAAGAAGAAGATACACAAGAAGAAAGAATAGAATCTGTTATTTGCAAG
AAATTTTCTCTAACGAAATGGCTAAGGTTGATGATTCTTTCTTTCATAGATTGGAAGAATCATTTTTAGTTGAAGAA
GATAAGAAACATGAAAGACATCCAATCTTCGGTAACATCGTTGATGAAGTTGCTTACCATGAAAAGTACCCAACAAT
CTATCATTTGAGAAAGAAATTGGTTGATTCAACTGATAAGGCAGATTTGAGATTGATATATTTGGCTTTAGCACATA
TGATCAAGTTTAGAGGTCATTTCTTGATCGAGGGTGACTTGAATCCAGATAATTCTGATGTTGATAAGTTGTTTATT
CAATTAGTTCAAACATATAATCAATTGTTTGAAGAAAATCCAATTAATGCTTCTGGTGTTGATGCTAAGGCAATCTT
GTCAGCAAGATTGTCTAAGTCAAGAAGATTGGAAAATTTGATCGCTCAATTACCAGGTGAAAAGAAAAATGGTTTGT
TCGGTAATTTGATCGCATTGTCTTTGGGTTTGACACCAAACTTCAAGTCAAACTTCGATTTGGCTGAAGATGCAAAG
TTGCAATTGTCTAAGGATACTTACGATGATGATTTGGATAATTTGTTGGCTCAAATTGGTGACCAATATGCAGATTT
GTTTTTGGCTGCTAAAAATTTGTCTGATGCTATCTTGTTGTCAGATATCTTGAGAGTTAACACTGAAATCACAAAGG
CTCCATTGTCTGCATCAATGATCAAGAGATACGATGAACATCATCAAGATTTGACTTTGTTGAAGGCATTGGTTAGA
CAACAATTACCAGAAAAGTACAAGGAAATTTTCTTTGATCAATCTAAAAATGGTTATGCTGGTTACATTGATGGTGG
TGCATCTCAAGAAGAATTCTACAAGTTTATTAAGCCAATCTTGGAAAAGATGGATGGTACAGAAGAATTGTTAGTTA
AATTGAACAGAGAAGATTTGTTAAGAAAACAAAGAACTTTCGATAACGGTTCTATCCCACATCAAATCCATTTGGGT
GAATTACATGCTATCTTGAGAAGACAAGAAGATTTCTACCCATTTTTAAAGGATAACAGAGAAAAGATTGAAAAGAT
TTTGACTTTTAGAATTCCATATTACGTTGGTCCATTAGCTCGTGGTAATTCTAGATTTGCATGGATGACTAGAAAGT
CAGAAGAAACTATCACACCATGGAATTTTGAAGAAGTTGTTGATAAAGGTGCTTCTGCACAATCTTTTATTGAAAGA
ATGACAAACTTCGATAAAAATTTGCCAAACGAAAAGGTTTTGCCAAAGCATTCATTGTTATATGAATACTTTACTGT
TTACAATGAATTGACAAAAGTTAAATATGTTACTGAGGGTATGAGAAAACCAGCATTTTTGTCTGGTGAACAAAAGA
AAGCAATCGTTGATTTGTTGTTTAAAACTAACAGAAAGGTTACAGTTAAACAATTGAAAGAAGATTACTTTAAGAAA
ATTGAATGTTTTGATTCTGTTGAAATTTCAGGTGTTGAAGATAGATTCAATGCTTCATTAGGTACTTACCATGATTT
GTTGAAGATTATTAAGGATAAAGATTTCTTGGATAATGAAGAAAATGAAGATATTTTAGAAGATATTGTTTTAACTT
TGACATTATTTGAAGATAGAGAAATGATCGAAGAAAGATTGAAGACATACGCTCATTTGTTCGATGATAAAGTTATG
AAGCAATTGAAGAGAAGAAGATACACTGGTTGGGGTAGATTGTCTAGAAAGTTGATTAATGGTATCAGAGATAAGCA
ATCTGGTAAAACAATCTTGGATTTCTTGAAGTCAGATGGTTTCGCAAACAGAAACTTCATGCAATTGATTCATGATG
ATTCATTGACTTTTAAAGAAGATATCCAAAAAGCTCAAGTTTCTGGTCAGGGTGACTCATTGCATGAACATATTGCT
AATTTGGCAGGTTCTCCAGCTATTAAGAAAGGTATCTTGCAAACAGTTAAGGTTGTTGATGAATTAGTTAAAGTTAT
GGGTAGACATAAGCCAGAAAACATCGTTATCGAAATGGCTAGAGAAAACCAAACTACACAAAAGGGTCAAAAGAATT
CAAGAGAAAGAATGAAGAGAATCGAAGAAGGTATTAAAGAATTGGGTTCTCAAATCTTGAAGGAACATCCAGTTGAA
AACACTCAATTGCAAAACGAAAAGTTGTACTTATACTACTTACAAAACGGTAGAGATATGTACGTTGATCAAGAATT
AGATATCAACAGATTGTCAGATTACGATGTTGATCATATCGTTCCACAATCATTTTTGAAGGATGATTCAATCGATA
ATAAGGTTTTGACAAGATCTGATAAGAACCGTGGTAAATCTGATAATGTTCCATCAGAAGAAGTTGTTAAGAAAATG
AAGAACTACTGGAGACAATTGTTAAATGCTAAGTTGATCACTCAAAGAAAGTTCGATAATTTGACAAAAGCTGAAAG
AGGTGGTTTGTCAGAATTAGATAAAGCAGGTTTTATTAAGAGACAATTAGTTGAAACTAGACAAATCACAAAGCATG
TTGCACAAATCTTGGATTCTAGAATGAACACTAAATATGATGAAAATGATAAATTAATTAGAGAAGTTAAAGTTATT
ACATTAAAATCTAAATTGGTTTCAGATTTTAGAAAAGATTTTCAATTCTACAAAGTTAGAGAAATTAATAACTATCA
TCATGCTCATGATGCATACTTGAATGCTGTTGTTGGTACTGCATTGATTAAGAAATACCCAAAGTTGGAATCTGAAT
TCGTTTACGGTGACTACAAGGTTTACGATGTTAGAAAGATGATCGCTAAGTCAGAACAAGAAATCGGTAAAGCTACA
GCAAAGTATTTCTTTTATTCTAACATCATGAATTTCTTTAAAACTGAAATTACATTAGCTAACGGTGAAATCAGAAA
AAGACCATTGATCGAAACTAATGGTGAAACAGGTGAAATTGTTTGGGATAAAGGTAGAGATTTCGCAACTGTTAGAA
AGGTTTTGTCAATGCCACAAGTTAACATCGTTAAGAAAACTGAAGTTCAAACAGGTGGTTTTTCTAAGGAATCAATC
TTGCCAAAGAGAAACTCTGATAAGTTGATTGCTAGAAAGAAAGATTGGGATCCAAAGAAATATGGTGGTTTTGATTC
TCCAACTGTTGCTTACTCAGTTTTAGTTGTTGCAAAGGTTGAAAAGGGTAAATCTAAGAAATTGAAATCAGTTAAAG
AATTGTTAGGTATCACAATCATGGAAAGATCTTCATTCGAAAAGAATCCAATCGATTTCTTGGAAGCAAAGGGTTAC
AAGGAAGTTAAGAAAGATTTGATTATTAAGTTGCCAAAGTACTCTTTGTTCGAATTAGAAAACGGTAGAAAAAGAAT
GTTAGCTTCAGCTGGTGAATTGCAAAAGGGTAATGAATTGGCTTTGCCATCTAAGTACGTTAATTTCTTGTATTTGG

CATCTCATTACGAAAAGTTGAAGGGTTCACCAGAAGATAATGAACAAAAACAATTGTTCGTTGAACAACATAAGCAT
TATTTGGATGAAATTATTGAACAAATTTCTGAATTTTCAAAAAGAGTTATTTTGGCTGATGCAAATTTGGATAAGGT
TTTGTCTGCTTACAATAAGCATAGAGATAAGCCAATCAGAGAACAAGCAGAAAACATCATCCATTTGTTTACTTTGA
CAAATTTGGGTGCTCCAGCTGCTTTTAAATACTTCGATACTACAATCGATAGAAAAAGATACACTTCTACAAAGGAA
GTTTTGGATGCAACATTGATCCATCAATCAATCACTGGTTTGTATGAAACAAGAATTGATTTGTCTCAATTGGGTGG
TGACGGTCGACGGATCCCCGGGTTAATTAACAGTAAAGGAGAAGAACTTTTCACTGGAGTTGTCCCAATTCTTGTTG
AATTAGATGGTGATGTTAATGGGCACAAATTTTCTGTCAGTGGGGAGGGTGAAGGTGATGCAACATACGGAAAACTT
ACCCTTAAATTTATTTGCACTACTGGAAAACTACCTGTTCCATGGCCAACACTTGTCACTACTTTGACTTATGGTGT
TCAATGCTTTTCAAGATACCCAGATCATATGAAACAACATGACTTTTTCAAGAGTGCCATGCCCGAAGGTTATGTAC
AGGAAAGAACTATATTTTTCAAAGATGACGGGAACTACAAGACACGTGCTGAAGTCAAGTTTGAAGGTGATACCCTT
GTTAATAGAATCGAGTTAAAAGGTATTGATTTTAAAGAAGATGGAAACATTCTTGGACACAAATTGGAATACAACTA
TAACTCACACAATGTATACATCATGGCAGACAAACAAAAGAATGGAATCAAAGTTAACTTCAAAATTAGACACAACA
TTGAAGATGGAAGCGTTCAACTAGCAGACCATTATCAACAAAATACTCCAATTGGCGATGGCCCTGTCCTTTTACCA
GACAACCATTACCTGTCCACACAATCTGCCCTTTCGAAAGATCCCAACGAAAAGAGAGACCACATGGTCCTTCTTGA
GTTTGTAACAGCTGCTGGGATTACATTGGGCATGGATGAACTATACAAATCTAGGGCAGACCCAAAGAAAAAGAGGA
AAGTAGGTGGATCCAGAGCTGATCCTAAAAAGAAAAGAAAGGTCTGAATCTCATAAGAATGGTGGTGATTATATATC
TTATGTTATTAAGAATTCTCAAATTATTCTATATGAAAACACCGTAACTTGCTTCTCTCCTTGGTTTTACATAATGA
CATAATGCGATCGAAAACTAGAGGTACAGGTATTGCTGGATTGGCGAGAGTTTTTACCTTCTTTTCTGGCGTACAGC
TATCACCTTCTCGTTTGGTAAAATGAAAGAACATTTTGTTGTCTTAGCCAAATATTTAATCTATGAAGAAAACGGAG
TTTACCGGTAATTCTAAATAAAAGTTTGGTTAGGGATTGTGCCTCATAGAGAAGCAATTGGTACTCATCTTATTAAA
GTATTACTATAAACATTAGAAAGAGTCCCTGAGCGTTGCTAATGGGAAGCTATTCGCGCTTTTAGTAAATTATTAAA
ATACGCCAAAAATAAATGTAATCCGGATATACCTCTTCTTTTAACCTTCCGTTTAGCTTGCCTCGTCCCCGCCGGGT
CACCCGGCCAGCGACATGGAGGCCCAGAATACCCTCCTTGACAGTCTTGACGTGCGCAGCTCAGGGGCATGATGTGA
CTGTCGCCCGTACATTTAGCCCATACATCCCCATGTATAATCATTTGCATCCATACATTTTGATGGCCGCACGGCGC
GAAGCAAAAATTACGGCTCCTCGCTGCAGACCTGCGAGCAGGGAAACGCTCCCCTCACAGACGCGTTGAATTGTCCC
CACGCCGCGCCCCTGTAGAGAAATATAAAAGGTTAGGATTTGCCACTGAGGTTCTTCTTTCATATACTTCCTTTTAA
AATCTTGCTAGGATACAGTTCTCACATCACATCCGAACATAAACAACCATGACAGTCAACACTAAGACCTATAGTGA
GAGAGCAGAAACTCATGCCTCACCAGTAGCACAACGATTATTTCGATTAATGGAACTGAAGAAAACCAATTTATGTG
CATCAATTGATGTTGATACCACTAAGGAATTCCTTGAATTAATTGATAAATTGGGTCCTTATGTATGCTTAATCAAG
ACTCATATTGATATAATCAATGATTTTTCCTATGAATCCACTATTGAACCATTATTAGAACTTTCACGTAAACATCA
ATTTATGATTTTTGAAGATAGAAAATTTGCTGATATTGGTAATACCGTGAAGAAACAATATATTGGTGGAGTTTATA
AAATTAGTAGTTGGGCAGATATTACTAATGCTCATGGTGTCACTGGGAATGGAGTAGTTGAAGGATTAAAACAGGGA
GCTAAAGAAACCACCACCAACCAAGAGCCAAGAGGGTTATTGATGTTAGCTGAATTATCATCAGTGGGATCATTAGC
ATATGGAGAATATTCTCAAAAAACTGTTGAAATTGCTAAATCCGATAAGGAATTTGTTATTGGATTTATTGCCCAAC
GTGATATGGGTGGACAAGAAGAAGGATTTGATTGGCTTATTATGACACCTGGAGTTGGATTAGATGATAAAGGTGAT
GGATTAGGACAACAATATAGAACTGTTGATGAAGTTGTTAGCACTGGAACTGATATTATCATTGTTGGTAGAGGATT
GTTTGGTAAAGGAAGAGATCCAGATATTGAAGGTAAAAGGTATAGAGATGCTGGTTGGAATGCTTATTTGAAAAAGA
CTGGCCAATTATAATCAGTACTGACAATAAAAAGATTCTTGTTTTCAAGAACTTGTCATTTGTATAGTTTTTTTATA
TTGTAGTTGTTCTATTTTAATCAAATGTTAGCGTGATTTATATTTTTTTTCGCCTCGACATCATCTGCCCAGATGCG
AAGTTAAGTGCGCAGAAAGTAATATCATGCGTCAATCGTATGTGAATGCTGGTCGCTATACTGCTGTCGATTCGATA
CTAACGCCGCCATCCAGTGCTGTTCGTGTGCGCGTCCTGGGTGACACCGATTATTTAAAGCTGCAGCATACGATATA
TATACATGTGTATATATGTATACCTATGAATGTCAGTAAGTATGTATACGAACAGTATGATACTGAAGATGACAAGG
TAATGCATCATTCTATACGTGTCATTCTGAACGAGGCGCGCTTTCCTTTTTTCTTTTTGCTTTTTCTTTTTTTTTCT
CTTGAACTCGAGAAAAAAAATATAAAAGAGATGGAGGAACGGGAAAAAGTTAGTTGTGGTGATAGGTGGCAAGTGGT
ATTCCGTAAGAACAACAAGAAAAGCATTTCATATTATGGCTGAACTGAGCGAACAAGTGCAAAATTTAAGCATCAAC
GACAACAACGAGAATGGTTATGTTCCTCCTCACTTAAGAGGAAAACCAAGAAGTGCCAGAAATAACAGTAGCAACTA
CAATAACAACAACGGCGGCTACAACGGTGGCCGTGGCGGTGGCAGCTTCTTTAGCAACAACCGTCGTGGTGGTTACG
GCAACGGTGGTTTCTTCGGTGGAAACAACGGTGGCAGCAGATCTAACGGCCGTTCTGGTGGTAGATGGATCGATGGC
AAACATGTCCCAGCTCCAAGAAACGAAAAGGCCGAGATCGCCATATTTGGTGTCCCCGAGGATCCAAATTTCCAATC
TTCTGGTATTAACTTCGATAACTACGATGATATTCCAGTGGACGCCTCTGGTAAGGATGTTCCTGAACCAATCACAG
AATTTACCTCACCTCCATTGGACGGATTGTTATTGGAAAACATCAAATTGGCCCGTTTCACCAAGCCAACACCTGTG
CAAAAATACTCCGTCCCTATCGTTGCCAACGGCAGAGATTTGATGGCCTGTGCGCAGACCGGTTCTGGTAAGACTGG
TGGGTTTTTATTCCCAGTGTTGTCCGAATCATTTAAGACTGGACCATCTCCTCAACCAGAGTCTCAAGGCTCCTTTT
ACCAAAGAAAGGCCTACCCAACTGCTGTCATTA

(989 bp 3′ UTR to *HIS3* shown)

GFY-3465: (identical to GFY-3470, but the identity of the C-terminal NLS sequence has been changed to the following):

AGAGCTGCTAAAAGACCAAGAACTACT **(RAAKRPRTT)**

GFY-3443:
AGAGCTGCTAAAAGATCTAGAACTACT **(RAAKRSRTT)**

GFY-3444:
TTAGCTGCTAAAAGACCAAGAACTACT **(LAAKRPRTT)**

GFY-3445:
AGAGCTGCTAAAAGATTAAGAACTACT **(RAAKRLRTT)**

GFY-3446:
CAAGCTGCTAAAAGATTAAGAACTACT **(QAAKRLRTT)**

GFY-3447:
CAAGCTGCTAAAAGATCAAGAACTACT **(QAAKRSRTT)**

GFY-3466: (identical to GFY-3470, but the identity of the C-terminal NLS sequence has been changed to the following):

GCTCCAGCTAAAAGAGCAAGAACTTCT **(APAKRARTS)**

GFY-3449:
GCTCCAGCTAAAAGAGCAAGAACTACT **(APAKRARTT)**

GFY-3450:
GCTCCAGCTAAAAGAGCAAGAGCTTCT **(APAKRARAS)**

GFY-3451:
GCTCCAGCTAAAAGAGCAAGAGCTACT **(APAKRARAT)**

GFY-3467: (identical to GFY-3470, but the identity of the C-terminal NLS sequence has been changed to the following):

GCTGCAGCTAAAAGATCTTGGTCTATGGCTTTT **(AAAKRSWSMAF)**

GFY-3452:
GCTGCAGCTAGAAGATCTTGGTCTATGGCTTTT **(AAARRSWSMAF)**

GFY-3454:
GCTGCAGCTAAAAGATCTGTTTCTATGGCTTTT **(AAAKRSVSMAF)**

GFY-3455:
GCTGCAGCTAAAAGATCTTGGTCTATGGTTTTT **(AAAKRSWSMVF)**

GFY-3456:
GCTGCAGCTAAAAGATCTTGGTCTATGGCTTAT **(AAAKRSWSMAY)**

GFY-3468: (identical to GFY-3470, but the identity of the C-terminal NLS sequence has been changed to the following):

TTGGCTAAAATTTTGGGTGCTTTGGATATTAAT (LAKILGALDIN)

GFY-3469:
TTGTTGCAACAATTACTATTATTGCAAATTAAT (LLQQLLLLQIN)

GFY-3471:
TTGGCTAAAATTTTGGGTGCTTTGGATATTAATggtggaTTAGCAAAGATATTAGGAGCATTAGACATCAAC
(LAKILGALDINGGLAKILGALDIN)

GFY-3472:
TTGGCTAAAATTTTGGGTGCTTTGGATATTAATggtggaTCCAGAGCCGACCCCAAGAAAAAAAGAAAAGTG
(LAKILGALDINGGSRADPKKKRKV)
(The *CDC10(t)* has a missing base pair "A" 61 bases downstream of the STOP codon).

GFY-2758:
TCCAGAGCCGACCCCAAGAAAAAAAGAAAAGTGggtggaTTGGCTAAAATTTTGGGTGCTTTGGATATTAAT
(SRADPKKKRKVGGLAKILGALDIN)

GFY-3716:

prHIS3::***(u2)***::*prGAL1/10*::***NLS***[SV40]::***SpCas9***::***NLS***[SV40]::***Linker***::***eGFP***::***NES***[PKI-like]::*CDC10(t)*::*prMX*::***Kan***[R]::*MX(t)*::***(u2)***::*HIS3(t)*

(u2) 20 bp target and 3 bp PAM sequence

(992 bp 5′ UTR to *HIS3* shown)

GGGTCAGTTATTTCATCCAGATATAACCCGAGAGGAAACTTCTTAGCGTCTGTTTTCGTACCATAAGGCAGTTCATG
AGGTATATTTTCGTTATTGAAGCCCAGCTCGTGAATGCTTAATGCTGCTGAACTGGTGTCCATGTCGCCTAGGTACG
CAATCTCCACAGGCTGCAAAGGTTTTGTCTCAAGAGCAATGTTATTGTGCACCCCGTAATTGGTCAACAAGTTTAAT
CTGTGCTTGTCCACCAGCTCTGTCGTAACCTTCAGTTCATCGACTATCTGAAGAAATTTACTAGGAATAGTGCCATG
GTACAGCAACCGAGAATGGCAATTTCTACTCGGGTTCAGCAACGCTGCATAAACGCTGTTGGTGCCGTAGACATATT
CGAAGATAGGATTATCATTCATAAGTTTCAGAGCAATGTCCTTATTCTGGAACTTGGATTTATGGCTCTTTTGGTTT
AATTTCGCCTGATTCTTGATCTCCTTTAGCTTCTCGACGTGGGCCTTTTTCTTGCCATATGGATCCGCTGCACGGTC
CTGTTCCCTAGCATGTACGTGAGCGTATTTCCTTTTAAACCACGACGCTTTGTCTTCATTCAACGTTTCCCATTGTT
TTTTTCTACTATTGCTTTGCTGTGGGAAAAACTTATCGAAAGATGACGACTTTTTCTTAATTCTCGTTTTAAGAGCT
TGGTGAGCGCTAGGAGTCACTGCCAGGTATCGTTTGAACACGGCATTAGTCAGGGAAGTCATAACACAGTCCTTTCC
CGCAATTTTCTTTTTCTATTACTCTTGGCCTCCTCTAGTACACTCTATATTTTTTTATGCCTCGGTAATGATTTTCA
TTTTTTTTTTTCCACCTAGCGGATGACTCTTTTTTTTTCTTAGCGATTGGCATTATCACATAATGAATTATACATTA
TATAAAGTAATGTGATTTCTTCGAAGAATATACTAAAAAATGAGCAGGCAAGATAAACGAAGGCAAAG**GCTGTTCGT**
GTGCGCGTCCTGGGGACAGGTTATCAGCAACAACACAGTCATATCCATTCTCAATTAGCTCTACCACAGTGTGTGAA
CCAATGTATCCAGCACCACCTGTAACCAAAACAATTTTAGAAGTACTTTCACTTTGTAACTGAGCTGTCATTTATAT
TGAATTTTCAAAAATTCTTACTTTTTTTTTGGATGGACGCAAAGAAGTTTAATAATCATATTACATGGCATTACCAC
CATATACATATCCATATACATATCCATATCTAATCTTACTTATATGTTGTGGAAATGTAAAGAGCCCCATTATCTTA
GCCTAAAAAAACCTTCTCTTTGGAACTTTCAGTAATACGCTTAACTGCTCATTGCTATATTGAAGTACGGATTAGAA
GCCGCCGAGCGGGTGACAGCCCTCCGAAGGAAGACTCTCCTCCGTGCGTCCTCGTCTTCACCGGTCGCGTTCCTGAA

ACGCAGATGTGCCTCGCGCCGCACTGCTCCGAACAATAAAGATTCTACAATACTAGCTTTTATGGTTATGAAGAGGA
AAAATTGGCAGTAACCTGGCCCCACAAACCTTCAAATGAACGAATCAAATTAACAACCATAGGATGATAATGCGATT
AGTTTTTTAGCCTTATTTCTGGGGTAATTAATCAGCGAAGCGATGATTTTTGATCTATTAACAGATATATAAATGCA
AAAACTGCATAACCACTTTAACTAATACTTTCAACATTTTCGGTTTGTATTACTTCTTATTCAAATGTAATAAAAGT
ATCAACAAAAAATTGTTAATATACCTCTATACTTTAACGTCAAGGAGAAAAAACTATAATGTCAAGAGCTGATCCTA
AAAAGAAAAGAAAGGTTGATAAGAAATACTCTATCGGTTTGGATATTGGTACAAATTCAGTTGGTTGGGCAGTTATT
ACTGATGAATACAAGGTTCCATCTAAAAAGTTTAAAGTTTTGGGTAACACTGATAGACATTCTATTAAGAAAAATTT
GATTGGTGCTTTGTTATTTGATTCTGGTGAAACTGCTGAAGCAACAAGATTGAAAAGAACTGCAAGAAGAAGATACA
CAAGAAGAAAGAATAGAATCTGTTATTTGCAAGAAATTTTCTCTAACGAAATGGCTAAGGTTGATGATTCTTTCTTT
CATAGATTGGAAGAATCATTTTTAGTTGAAGAAGATAAGAAACATGAAAGACATCCAATCTTCGGTAACATCGTTGA
TGAAGTTGCTTACCATGAAAAGTACCCAACAATCTATCATTTGAGAAAGAAATTGGTTGATTCAACTGATAAGGCAG
ATTTGAGATTGATATATTTGGCTTTAGCACATATGATCAAGTTTAGAGGTCATTTCTTGATCGAGGGTGACTTGAAT
CCAGATAATTCTGATGTTGATAAGTTGTTTATTCAATTAGTTCAAACATATAATCAATTGTTTGAAGAAAATCCAAT
TAATGCTTCTGGTGTTGATGCTAAGGCAATCTTGTCAGCAAGATTGTCTAAGTCAAGAAGATTGGAAAATTTGATCG
CTCAATTACCAGGTGAAAAGAAAAATGGTTTGTTCGGTAATTTGATCGCATTGTCTTTGGGTTTGACACCAAACTTC
AAGTCAAACTTCGATTTGGCTGAAGATGCAAAGTTGCAATTGTCTAAGGATACTTACGATGATGATTTGGATAATTT
GTTGGCTCAAATTGGTGACCAATATGCAGATTTGTTTTTGGCTGCTAAAAATTTGTCTGATGCTATCTTGTTGTCAG
ATATCTTGAGAGTTAACACTGAAATCACAAAGGCTCCATTGTCTGCATCAATGATCAAGAGATACGATGAACATCAT
CAAGATTTGACTTTGTTGAAGGCATTGGTTAGACAACAATTACCAGAAAAGTACAAGGAAATTTTCTTTGATCAATC
TAAAAATGGTTATGCTGGTTACATTGATGGTGGTGCATCTCAAGAAGAATTCTACAAGTTTATTAAGCCAATCTTGG
AAAAGATGGATGGTACAGAAGAATTGTTAGTTAAATTGAACAGAGAAGATTTGTTAAGAAAACAAAGAACTTTCGAT
AACGGTTCTATCCCACATCAAATCCATTTGGGTGAATTACATGCTATCTTGAGAAGACAAGAAGATTTCTACCCATT
TTTAAAGGATAACAGAGAAAAGATTGAAAAGATTTTGACTTTTAGAATTCCATATTACGTTGGTCCATTAGCTCGTG
GTAATTCTAGATTTGCATGGATGACTAGAAAGTCAGAAGAAACTATCACACCATGGAATTTTGAAGAAGTTGTTGAT
AAAGGTGCTTCTGCACAATCTTTTATTGAAAGAATGACAAACTTCGATAAAAATTTGCCAAACGAAAAGGTTTTGCC
AAAGCATTCATTGTTATATGAATACTTTACTGTTTACAATGAATTGACAAAAGTTAAATATGTTACTGAGGGTATGA
GAAAACCAGCATTTTTGTCTGGTGAACAAAAGAAAGCAATCGTTGATTTGTTGTTTAAAACTAACAGAAAGGTTACA
GTTAAACAATTGAAAGAAGATTACTTTAAGAAAATTGAATGTTTTGATTCTGTTGAAATTTCAGGTGTTGAAGATAG
ATTCAATGCTTCATTAGGTACTTACCATGATTTGTTGAAGATTATTAAGGATAAAGATTTCTTGGATAATGAAGAAA
ATGAAGATATTTTAGAAGATATTGTTTTAACTTTGACATTATTTGAAGATAGAGAAATGATCGAAGAAAGATTGAAG
ACATACGCTCATTTGTTCGATGATAAAGTTATGAAGCAATTGAAGAGAAGAAGATACACTGGTTGGGGTAGATTGTC
TAGAAAGTTGATTAATGGTATCAGAGATAAGCAATCTGGTAAAACAATCTTGGATTTCTTGAAGTCAGATGGTTTCG
CAAACAGAAACTTCATGCAATTGATTCATGATGATTCATTGACTTTTAAAGAAGATATCCAAAAAGCTCAAGTTTCT
GGTCAGGGTGACTCATTGCATGAACATATTGCTAATTTGGCAGGTTCTCCAGCTATTAAGAAAGGTATCTTGCAAAC
AGTTAAGGTTGTTGATGAATTAGTTAAAGTTATGGGTAGACATAAGCCAGAAAACATCGTTATCGAAATGGCTAGAG
AAAACCAAACTACACAAAAGGGTCAAAAGAATTCAAGAGAAAGAATGAAGAGAATCGAAGAAGGTATTAAAGAATTG
GGTTCTCAAATCTTGAAGGAACATCCAGTTGAAAACACTCAATTGCAAAACGAAAAGTTGTACTTATACTACTTACA
AAACGGTAGAGATATGTACGTTGATCAAGAATTAGATATCAACAGATTGTCAGATTACGATGTTGATCATATCGTTC
CACAATCATTTTTGAAGGATGATTCAATCGATAATAAGGTTTTGACAAGATCTGATAAGAACCGTGGTAAATCTGAT
AATGTTCCATCAGAAGAAGTTGTTAAGAAAATGAAGAACTACTGGAGACAATTGTTAAATGCTAAGTTGATCACTCA
AAGAAAGTTCGATAATTTGACAAAAGCTGAAAGAGGTGGTTTGTCAGAATTAGATAAAGCAGGTTTTATTAAGAGAC
AATTAGTTGAAACTAGACAAATCACAAAGCATGTTGCACAAATCTTGGATTCTAGAATGAACACTAAATATGATGAA
AATGATAAATTAATTAGAGAAGTTAAAGTTATTACATTAAAATCTAAATTGGTTTCAGATTTTAGAAAAGATTTTCA
ATTCTACAAAGTTAGAGAAATTAATAACTATCATCATGCTCATGATGCATACTTGAATGCTGTTGTTGGTACTGCAT
TGATTAAGAAATACCCAAAGTTGGAATCTGAATTCGTTTACGGTGACTACAAGGTTTACGATGTTAGAAAGATGATC
GCTAAGTCAGAACAAGAAATCGGTAAAGCTACAGCAAAGTATTTCTTTTATTCTAACATCATGAATTTCTTTAAAAC
TGAAATTACATTAGCTAACGGTGAAATCAGAAAAAGACCATTGATCGAAACTAATGGTGAAACAGGTGAAATTGTTT
GGGATAAAGGTAGAGATTTCGCAACTGTTAGAAAGGTTTTGTCAATGCCACAAGTTAACATCGTTAAGAAAACTGAA
GTTCAAACAGGTGGTTTTTCTAAGGAATCAATCTTGCCAAAGAGAAACTCTGATAAGTTGATTGCTAGAAAGAAAGA
TTGGGATCCAAAGAAATATGGTGGTTTTGATTCTCCAACTGTTGCTTACTCAGTTTTAGTTGTTGCAAAGGTTGAAA
AGGGTAAATCTAAGAAATTGAAATCAGTTAAAGAATTGTTAGGTATCACAATCATGGAAAGATCTTCATTCGAAAAG
AATCCAATCGATTTCTTGGAAGCAAAGGGTTACAAGGAAGTTAAGAAAGATTTGATTATTAAGTTGCCAAAGTACTC
TTTGTTCGAATTAGAAAACGGTAGAAAAAGAATGTTAGCTTCAGCTGGTGAATTGCAAAAGGGTAATGAATTGGCTT
TGCCATCTAAGTACGTTAATTTCTTGTATTTGGCATCTCATTACGAAAAGTTGAAGGGTTCACCAGAAGATAATGAA
CAAAAACAATTGTTCGTTGAACAACATAAGCATTATTTGGATGAAATTATTGAACAAATTTCTGAATTTTCAAAAAG
AGTTATTTTGGCTGATGCAAATTTGGATAAGGTTTTGTCTGCTTACAATAAGCATAGAGATAAGCCAATCAGAGAAC
AAGCAGAAAACATCATCCATTTGTTTACTTTGACAAATTTGGGTGCTCCAGCTGCTTTTAAATACTTCGATACTACA

ATCGATAGAAAAAGATACACTTCTACAAAGGAAGTTTTGGATGCAACATTGATCCATCAATCAATCACTGGTTTGTA
TGAAACAAGAATTGATTTGTCTCAATTGGGTGGTGACTCTAGGGCAGACCCAAAGAAAAAGAGGAAAGTAAGTGGTT
CTGGATCAGGTCGACGGATCCCCGGGTTAATTAACAGTAAAGGAGAAGAACTTTTCACTGGAGTTGTCCCAATTCTT
GTTGAATTAGATGGTGATGTTAATGGGCACAAATTTTCTGTCAGTGGGGAGGGTGAAGGTGATGCAACATACGGAAA
ACTTACCCTTAAATTTATTTGCACTACTGGAAAACTACCTGTTCCATGGCCAACACTTGTCACTACTTTGACTTATG
GTGTTCAATGCTTTTCAAGATACCCAGATCATATGAAACAACATGACTTTTTCAAGAGTGCCATGCCCGAAGGTTAT
GTACAGGAAAGAACTATATTTTTCAAAGATGACGGGAACTACAAGACACGTGCTGAAGTCAAGTTTGAAGGTGATAC
CCTTGTTAATAGAATCGAGTTAAAAGGTATTGATTTTAAAGAAGATGGAAACATTCTTGGACACAAATTGGAATACA
ACTATAACTCACACAATGTATACATCATGGCAGACAAACAAAAGAATGGAATCAAAGTTAACTTCAAAATTAGACAC
AACATTGAAGATGGAAGCGTTCAACTAGCAGACCATTATCAACAAAATACTCCAATTGGCGATGGCCCTGTCCTTTT
ACCAGACAACCATTACCTGTCCACACAATCTGCCCTTTCGAAAGATCCCAACGAAAAGAGAGACCACATGGTCCTTC
TTGAGTTTGTAACAGCTGCTGGGATTACATTGGGCATGGATGAACTATACAAATTGGCTAAAATTTTGGGTGCTTTG
GATATTAATTGAATCTCATAAGAATGGTGGTGATTATATATCTTATGTTATTAAGAATTCTCAAATTATTCTATATG
AAAACACCGTAACTTGCTTCTCTCCTTGGTTTTACATAATGACATAATGCGATCGAAAACTAGAGGTACAGGTATTG
CTGGATTGGCGAGAGTTTTTACCTTCTTTTCTGGCGTACAGCTATCACCTTCTCGTTTGGTAAAATGAAAGAACATT
TTGTTGTCTTAGCCAAATATTTAATCTATGAAGAAAACGGAGTTTACCGGTAATTCTAAATAAAAGTTTGGTTAGGG
ATTGTGCCTCATAGAGAAGCAATTGGTACTCATCTTATTAAAGTATTACTATAAACATTAGAAAGAGTCCCTGAGCG
TTGCTAATGGGAAGCTATTCGCGCTTTTAGTAAATTATTAAAATACGCCAAAAATAAATGTAATCCGGATATACCTC
TTCTTTTAACCTTCCGTTTAGCTTGCCTCGTCCCCGCCGGGTCACCCGGCCAGCGACATGGAGGCCCAGAATACCCT
CCTTGACAGTCTTGACGTGCGCAGCTCAGGGGCATGATGTGACTGTCGCCCGTACATTTAGCCCATACATCCCCATG
TATAATCATTTGCATCCATACATTTTGATGGCCGCACGGCGCGAAGCAAAAATTACGGCTCCTCGCTGCAGACCTGC
GAGCAGGGAAACGCTCCCCTCACAGACGCGTTGAATTGTCCCCACGCCGCGCCCCTGTAGAGAAATATAAAAGGTTA
GGATTTGCCACTGAGGTTCTTCTTTCATATACTTCCTTTTAAAATCTTGCTAGGATACAGTTCTCACATCACATCCG
AACATAAACAACCATGGGTAAGGAAAAGACTCACGTTTCGAGGCCGCGATTAAATTCCAACATGGATGCTGATTTAT
ATGGGTATAAATGGGCTCGCGATAATGTCGGGCAATCAGGTGCGACAATCTATCGATTGTATGGGAAGCCCGATGCG
CCAGAGTTGTTTCTGAAACATGGCAAAGGTAGCGTTGCCAATGATGTTACAGATGAGATGGTCAGACTAAACTGGCT
GACGGAATTTATGCCTCTTCCGACCATCAAGCATTTTATCCGTACTCCTGATGATGCATGGTTACTCACCACTGCGA
TCCCCGGCAAAACAGCATTCCAGGTATTAGAAGAATATCCTGATTCAGGTGAAAATATTGTTGATGCGCTGGCAGTG
TTCCTGCGCCGGTTGCATTCGATTCCTGTTTGTAATTGTCCTTTTAACAGCGATCGCGTATTTCGTCTCGCTCAGGC
GCAATCACGAATGAATAACGGTTTGGTTGATGCGAGTGATTTTGATGACGAGCGTAATGGCTGGCCTGTTGAACAAG
TCTGGAAAGAAATGCATAAGCTTTTGCCATTCTCACCGGATTCAGTCGTCACTCATGGTGATTTCTCACTTGATAAC
CTTATTTTTGACGAGGGGAAATTAATAGGTTGTATTGATGTTGGACGAGTCGGAATCGCAGACCGATACCAGGATCT
TGCCATCCTATGGAACTGCCTCGGTGAGTTTTCTCCTTCATTACAGAAACGGCTTTTTCAAAAATATGGTATTGATA
ATCCTGATATGAATAAATTGCAGTTTCATTTGATGCTCGATGAGTTTTTCTAATCAGTACTGACAATAAAAAGATTC
TTGTTTTCAAGAACTTGTCATTTGTATAGTTTTTTTATATTGTAGTTGTTCTATTTTAATCAAATGTTAGCGTGATT
TATATTTTTTTTCGCCTCGACATCATCTGCCCAGATGCGAAGTTAAGTGCGCAGAAAGTAATATCATGCGTCAATCG
TATGTGAATGCTGGTCGCTATACTGCTGTCGATTCGATACTAACGCCGCCATCCAGTGCTGTTCGTGTGCGCGTCCT
GGGTGACACCGATTATTTAAAGCTGCAGCATACGATATATATACATGTGTATATATGTATACCTATGAATGTCAGTA
AGTATGTATACGAACAGTATGATACTGAAGATGACAAGGTAATGCATCATTCTATACGTGTCATTCTGAACGAGGCG
CGCTTTCCTTTTTTCTTTTTGCTTTTTCTTTTTTTTTCTCTTGAACTCGAGAAAAAAAATATAAAAGAGATGGAGGA
ACGGGAAAAAGTTAGTTGTGGTGATAGGTGGCAAGTGGTATTCCGTAAGAACAACAAGAAAAGCATTTCATATTATG
GCTGAACTGAGCGAACAAGTGCAAAATTTAAGCATCAACGACAACAACGAGAATGGTTATGTTCCTCCTCACTTAAG
AGGAAAACCAAGAAGTGCCAGAAATAACAGTAGCAACTACAATAACAACAACGGCGGCTACAACGGTGGCCGTGGCG
GTGGCAGCTTCTTTAGCAACAACCGTCGTGGTGGTTACGGCAACGGTGGTTTCTTCGGTGGAAACAACGGTGGCAGC
AGATCTAACGGCCGTTCTGGTGGTAGATGGATCGATGGCAAACATGTCCCAGCTCCAAGAAACGAAAAGGCCGAGAT
CGCCATATTTGGTGTCCCCGAGGATCCAAATTTCCAATCTTCTGGTATTAACTTCGATAACTACGATGATATTCCAG
TGGACGCCTCTGGTAAGGATGTTCCTGAACCAATCACAGAATTTACCTCACCTCCATTGGACGGATTGTTATTGGAA
AACATCAAATTGGCCCGTTTCACCAAGCCAACACCTGTGCAAAAATACTCCGTCCCTATCGTTGCCAACGGCAGAGA
TTTGATGGCCTGTGCGCAGACCGGTTCTGGTAAGACTGGTGGGTTTTTATTCCCAGTGTTGTCCGAATCATTTAAGA
CTGGACCATCTCCTCAACCAGAGTCTCAAGGCTCCTTTTACCAAAGAAAGGCCTACCCAACTGCTGTCATTA
(989 bp 3′ UTR to *HIS3* shown)

GFY-3717: (Identical to GFY-3716, but with the C-terminal NES changed to the following):

TTGTTGCAACAATTACTATTATTGCAAATTAAT (LLQQLLLLQIN)

GFY-3435:

prHIS3::***(u2)***::*prGAL1/10*::***SpCas9***::***eGFP***::***(NLS$^{Class2-I}$)***::*CDC10(t)*::*prCCW12*::
SpHIS5::*MX(t)*::***(u2)***::*HIS3(t)*

(u2) 20 bp target and 3 bp PAM sequence

(992 bp 5′ UTR to *HIS3* shown)

GGGTCAGTTATTTCATCCAGATATAACCCGAGAGGAAACTTCTTAGCGTCTGTTTTCGTACCATAAGGCAGTTCATG
AGGTATATTTTCGTTATTGAAGCCCAGCTCGTGAATGCTTAATGCTGCTGAACTGGTGTCCATGTCGCCTAGGTACG
CAATCTCCACAGGCTGCAAAGGTTTTGTCTCAAGAGCAATGTTATTGTGCACCCCGTAATTGGTCAACAAGTTTAAT
CTGTGCTTGTCCACCAGCTCTGTCGTAACCTTCAGTTCATCGACTATCTGAAGAAATTTACTAGGAATAGTGCCATG
GTACAGCAACCGAGAATGGCAATTTCTACTCGGGTTCAGCAACGCTGCATAAACGCTGTTGGTGCCGTAGACATATT
CGAAGATAGGATTATCATTCATAAGTTTCAGAGCAATGTCCTTATTCTGGAACTTGGATTTATGGCTCTTTTGGTTT
AATTTCGCCTGATTCTTGATCTCCTTTAGCTTCTCGACGTGGGCCTTTTTCTTGCCATATGGATCCGCTGCACGGTC
CTGTTCCCTAGCATGTACGTGAGCGTATTTCCTTTTAAACCACGACGCTTTGTCTTCATTCAACGTTTCCCATTGTT
TTTTTCTACTATTGCTTTGCTGTGGGAAAAACTTATCGAAAGATGACGACTTTTTCTTAATTCTCGTTTTAAGAGCT
TGGTGAGCGCTAGGAGTCACTGCCAGGTATCGTTTGAACACGGCATTAGTCAGGGAAGTCATAACACAGTCCTTTCC
CGCAATTTTCTTTTTCTATTACTCTTGGCCTCCTCTAGTACACTCTATATTTTTTTATGCCTCGGTAATGATTTTCA
TTTTTTTTTTTCCACCTAGCGGATGACTCTTTTTTTTTCTTAGCGATTGGCATTATCACATAATGAATTATACATTA
TATAAAGTAATGTGATTTCTTCGAAGAATATACTAAAAAATGAGCAGGCAAGATAAACGAAGGCAAAG**GCTGTTCGT**
GTGCGCGTCCTGGGGACAGGTTATCAGCAACAACACAGTCATATCCATTCTCAATTAGCTCTACCACAGTGTGTGAA
CCAATGTATCCAGCACCACCTGTAACCAAAACAATTTTAGAAGTACTTTCACTTTGTAACTGAGCTGTCATTTATAT
TGAATTTTCAAAAATTCTTACTTTTTTTTTGGATGGACGCAAAGAAGTTTAATAATCATATTACATGGCATTACCAC
CATATACATATCCATATACATATCCATATCTAATCTTACTTATATGTTGTGGAAATGTAAAGAGCCCCATTATCTTA
GCCTAAAAAAACCTTCTCTTTGGAACTTTCAGTAATACGCTTAACTGCTCATTGCTATATTGAAGTACGGATTAGAA
GCCGCCGAGCGGGTGACAGCCCTCCGAAGGAAGACTCTCCTCCGTGCGTCCTCGTCTTCACCGGTCGCGTTCCTGAA
ACGCAGATGTGCCTCGCGCCGCACTGCTCCGAACAATAAAGATTCTACAATACTAGCTTTTATGGTTATGAAGAGGA
AAAATTGGCAGTAACCTGGCCCCACAAACCTTCAAATGAACGAATCAAATTAACAACCATAGGATGATAATGCGATT
AGTTTTTTAGCCTTATTTCTGGGGTAATTAATCAGCGAAGCGATGATTTTTGATCTATTAACAGATATATAAATGCA
AAAACTGCATAACCACTTTAACTAATACTTTCAACATTTTCGGTTTGTATTACTTCTTATTCAAATGTAATAAAAGT
ATCAACAAAAAATTGTTAATATACCTCTATACTTTAACGTCAAGGAGAAAAAACTATA**ATGGATAAGAAATACTCTA**
TCGGTTTGGATATTGGTACAAATTCAGTTGGTTGGGCAGTTATTACTGATGAATACAAGGTTCCATCTAAAAAGTTT
AAAGTTTTGGGTAACACTGATAGACATTCTATTAAGAAAAATTTGATTGGTGCTTTGTTATTTGATTCTGGTGAAAC
TGCTGAAGCAACAAGATTGAAAAGAACTGCAAGAAGAAGATACACAAGAAGAAAGAATAGAATCTGTTATTTGCAAG
AAATTTTCTCTAACGAAATGGCTAAGGTTGATGATTCTTTCTTTCATAGATTGGAAGAATCATTTTTAGTTGAAGAA
GATAAGAAACATGAAAGACATCCAATCTTCGGTAACATCGTTGATGAAGTTGCTTACCATGAAAAGTACCCAACAAT
CTATCATTTGAGAAAGAAATTGGTTGATTCAACTGATAAGGCAGATTTGAGATTGATATATTTGGCTTTAGCACATA
TGATCAAGTTTAGAGGTCATTTCTTGATCGAGGGTGACTTGAATCCAGATAATTCTGATGTTGATAAGTTGTTTATT
CAATTAGTTCAAACATATAATCAATTGTTTGAAGAAAATCCAATTAATGCTTCTGGTGTTGATGCTAAGGCAATCTT
GTCAGCAAGATTGTCTAAGTCAAGAAGATTGGAAAATTTGATCGCTCAATTACCAGGTGAAAAGAAAAATGGTTTGT
TCGGTAATTTGATCGCATTGTCTTTGGGTTTGACACCAAACTTCAAGTCAAACTTCGATTTGGCTGAAGATGCAAAG
TTGCAATTGTCTAAGGATACTTACGATGATGATTTGGATAATTTGTTGGCTCAAATTGGTGACCAATATGCAGATTT
GTTTTTGGCTGCTAAAAATTTGTCTGATGCTATCTTGTTGTCAGATATCTTGAGAGTTAACACTGAAATCACAAAGG
CTCCATTGTCTGCATCAATGATCAAGAGATACGATGAACATCATCAAGATTTGACTTTGTTGAAGGCATTGGTTAGA
CAACAATTACCAGAAAAGTACAAGGAAATTTTCTTTGATCAATCTAAAAATGGTTATGCTGGTTACATTGATGGTGG
TGCATCTCAAGAAGAATTCTACAAGTTTATTAAGCCAATCTTGGAAAAGATGGATGGTACAGAAGAATTGTTAGTTA
AATTGAACAGAGAAGATTTGTTAAGAAAACAAAGAACTTTCGATAACGGTTCTATCCCACATCAAATCCATTTGGGT
GAATTACATGCTATCTTGAGAAGACAAGAAGATTTCTACCCATTTTTAAAGGATAACAGAGAAAAGATTGAAAAGAT
TTTGACTTTTAGAATTCCATATTACGTTGGTCCATTAGCTCGTGGTAATTCTAGATTTGCATGGATGACTAGAAAGT
CAGAAGAAACTATCACACCATGGAATTTTGAAGAAGTTGTTGATAAAGGTGCTTCTGCACAATCTTTTATTGAAAGA
ATGACAAACTTCGATAAAAATTTGCCAAACGAAAAGGTTTTGCCAAAGCATTCATTGTTATATGAATACTTTACTGT
TTACAATGAATTGACAAAAGTTAAATATGTTACTGAGGGTATGAGAAAACCAGCATTTTTGTCTGGTGAACAAAAGA
AAGCAATCGTTGATTTGTTGTTTAAAACTAACAGAAAGGTTACAGTTAAACAATTGAAAGAAGATTACTTTAAGAAA

**ATTGAATGTTTTGATTCTGTTGAAATTTCAGGTGTTGAAGATAGATTCAATGCTTCATTAGGTACTTACCATGATTT
GTTGAAGATTATTAAGGATAAAGATTTCTTGGATAATGAAGAAAATGAAGATATTTTAGAAGATATTGTTTTAACTT
TGACATTATTTGAAGATAGAGAAATGATCGAAGAAAGATTGAAGACATACGCTCATTTGTTCGATGATAAAGTTATG
AAGCAATTGAAGAGAAGAAGATACACTGGTTGGGGTAGATTGTCTAGAAAGTTGATTAATGGTATCAGAGATAAGCA
ATCTGGTAAAACAATCTTGGATTTCTTGAAGTCAGATGGTTTCGCAAACAGAAACTTCATGCAATTGATTCATGATG
ATTCATTGACTTTTAAAGAAGATATCCAAAAAGCTCAAGTTTCTGGTCAGGGTGACTCATTGCATGAACATATTGCT
AATTTGGCAGGTTCTCCAGCTATTAAGAAAGGTATCTTGCAAACAGTTAAGGTTGTTGATGAATTAGTTAAAGTTAT
GGGTAGACATAAGCCAGAAAACATCGTTATCGAAATGGCTAGAGAAAACCAAACTACACAAAAGGGTCAAAAGAATT
CAAGAGAAAGAATGAAGAGAATCGAAGAAGGTATTAAAGAATTGGGTTCTCAAATCTTGAAGGAACATCCAGTTGAA
AACACTCAATTGCAAAACGAAAAGTTGTACTTATACTACTTACAAAACGGTAGAGATATGTACGTTGATCAAGAATT
AGATATCAACAGATTGTCAGATTACGATGTTGATCATATCGTTCCACAATCATTTTTGAAGGATGATTCAATCGATA
ATAAGGTTTTGACAAGATCTGATAAGAACCGTGGTAAATCTGATAATGTTCCATCAGAAGAAGTTGTTAAGAAAATG
AAGAACTACTGGAGACAATTGTTAAATGCTAAGTTGATCACTCAAAGAAAGTTCGATAATTTGACAAAAGCTGAAAG
AGGTGGTTTGTCAGAATTAGATAAAGCAGGTTTTATTAAGAGACAATTAGTTGAAACTAGACAAATCACAAAGCATG
TTGCACAAATCTTGGATTCTAGAATGAACACTAAATATGATGAAAATGATAAATTAATTAGAGAAGTTAAAGTTATT
ACATTAAAATCTAAATTGGTTTCAGATTTTAGAAAAGATTTTCAATTCTACAAAGTTAGAGAAATTAATAACTATCA
TCATGCTCATGATGCATACTTGAATGCTGTTGTTGGTACTGCATTGATTAAGAAATACCCAAAGTTGGAATCTGAAT
TCGTTTACGGTGACTACAAGGTTTACGATGTTAGAAAGATGATCGCTAAGTCAGAACAAGAAATCGGTAAAGCTACA
GCAAAGTATTTCTTTTATTCTAACATCATGAATTTCTTTAAAACTGAAATTACATTAGCTAACGGTGAAATCAGAAA
AAGACCATTGATCGAAACTAATGGTGAAACAGGTGAAATTGTTTGGGATAAAGGTAGAGATTTCGCAACTGTTAGAA
AGGTTTTGTCAATGCCACAAGTTAACATCGTTAAGAAAACTGAAGTTCAAACAGGTGGTTTTTCTAAGGAATCAATC
TTGCCAAAGAGAAACTCTGATAAGTTGATTGCTAGAAAGAAAGATTGGGATCCAAAGAAATATGGTGGTTTTGATTC
TCCAACTGTTGCTTACTCAGTTTTAGTTGTTGCAAAGGTTGAAAAGGGTAAATCTAAGAAATTGAAATCAGTTAAAG
AATTGTTAGGTATCACAATCATGGAAAGATCTTCATTCGAAAAGAATCCAATCGATTTCTTGGAAGCAAAGGGTTAC
AAGGAAGTTAAGAAAGATTTGATTATTAAGTTGCCAAAGTACTCTTTGTTCGAATTAGAAAACGGTAGAAAAAGAAT
GTTAGCTTCAGCTGGTGAATTGCAAAAGGGTAATGAATTGGCTTTGCCATCTAAGTACGTTAATTTCTTGTATTTGG
CATCTCATTACGAAAAGTTGAAGGGTTCACCAGAAGATAATGAACAAAAACAATTGTTCGTTGAACAACATAAGCAT
TATTTGGATGAAATTATTGAACAAATTTCTGAATTTTCAAAAAGAGTTATTTTGGCTGATGCAAATTTGGATAAGGT
TTTGTCTGCTTACAATAAGCATAGAGATAAGCCAATCAGAGAACAAGCAGAAAACATCATCCATTTGTTTACTTTGA
CAAATTTGGGTGCTCCAGCTGCTTTTAAATACTTCGATACTACAATCGATAGAAAAAGATACACTTCTACAAAGGAA
GTTTTGGATGCAACATTGATCCATCAATCAATCACTGGTTTGTATGAAACAAGAATTGATTTGTCTCAATTGGGTGG
TGACGGTCGACGGATCCCCGGGTTAATTAACAGTAAAGGAGAAGAACTTTTCACTGGAGTTGTCCCAATTCTTGTTG
AATTAGATGGTGATGTTAATGGGCACAAATTTTCTGTCAGTGGGGAGGGTGAAGGTGATGCAACATACGGAAAACTT
ACCCTTAAATTTATTTGCACTACTGGAAAACTACCTGTTCCATGGCCAACACTTGTCACTACTTTGACTTATGGTGT
TCAATGCTTTTCAAGATACCCAGATCATATGAAACAACATGACTTTTTCAAGAGTGCCATGCCCGAAGGTTATGTAC
AGGAAAGAACTATATTTTTCAAAGATGACGGGAACTACAAGACACGTGCTGAAGTCAAGTTTGAAGGTGATACCCTT
GTTAATAGAATCGAGTTAAAAGGTATTGATTTTAAAGAAGATGGAAACATTCTTGGACACAAATTGGAATACAACTA
TAACTCACACAATGTATACATCATGGCAGACAAACAAAAGAATGGAATCAAAGTTAACTTCAAAATTAGACACAACA
TTGAAGATGGAAGCGTTCAACTAGCAGACCATTATCAACAAAATACTCCAATTGGCGATGGCCCTGTCCTTTTACCA
GACAACCATTACCTGTCCACACAATCTGCCCTTTCGAAAGATCCCAACGAAAAGAGAGACCACATGGTCCTTCTTGA
GTTTGTAACAGCTGCTGGGATTACATTGGGCATGGATGAACTATACAAAAGAGCTGCTAAAAGACCAAGAACTACTT
GA**ATCTCATAAGAATGGTGGTGATTATATATCTTATGTTATTAAGAATTCTCAAATTATTCTATATGAAAACACCGT
AACTTGCTTCTCTCCTTGGTTTTACATAATGACATAATGCGATCGAAAACTAGAGGTACAGGTATTGCTGGATTGGC
GAGAGTTTTTACCTTCTTTTCTGGCGTACAGCTATCACCTTCTCGTTTGGTAAAATGAAAGAACATTTTGTTGTCTT
AGCCAAATATTTAATCTATGAAGAAAACGGAGTTTACCGGTAATTCTAAATAAAAGTTTGGTTAGGGATTGTGCCTC
ATAGAGAAGCAATTGGTACTCATCTTATTAAAGTATTACTATAAACATTAGAAAGAGTCCCTGAGCGTTGCTAATGG
GAAGCTATTCGCGCTTTTAGTAAATTATTAAAATACGCCAAAAATAAATGTAATCCGGATATACCTCTTCTTTTAAC
CTTCCCAAAGCAAAATAAAAGAAACTTAATACGTTATGCCGTAATGAAGGGCTACCAAAAACGATAATCTCAACTGT
AAACAGGTACAATGCGGACCCTTTTGCCACAAAACATACATCATTCATTGCCGGAAAAAGAAAGAAGTGAAGACAGC
AGTGCAGCCAGCCATGTTGCGCCAATCTAATTATAGATGCTGGTGCCCTGAGGATGTATCTGGAGCCAGCCATGGCA
TCATGCGCTACCGCCGGATGTAAAATCCGACACGCAAAAGAAAACCTTCGAGGTTGCGCACTTCGCCCACCCATGAA
CCACACGGTTAGTCCAAAAGGGGCAGTTCAGATTCCAGATGCGGGAATTAGCTTGCTGCCACCCTCACCTCACTAAC
GCTGCGGTGTGCGGATACTTCATGCTATTTATAGACGCGCGTGTCGGAATCAGCACGCGCAAGAACCAAATGGGAAA
ATCGGAATGGGTCCAGAACTGCTTTGAGTGCTGGCTATTGGCGTCTGATTTCCGTTTTGGGAATCCTTTGCCGCGCG
CCCCTCTCAAAACTCCGCACAAGTCCCAGAAAGCGGGAAAGAATAAAACGCCACCAAAAAAAAAAAAATAAAAGCC
AATCCTCGAAGCGTGGGTGGTAGGCCCTGGATTATCCCGTACAAGTATTTCTCAGGAGTAAAAAAACCGTTTGTTTT
GGAATTCCCCATTTCGCGGCCACCTACGCCGCTATCTTTGCAACAACTATCTGCGATAACTCAGCAAATTTTGCATA

TTCGTGTTGCAGTATTGCGATAATGGGAGTCTTACTTCCAACATAACGGCAGAAAGAAATGTGAGAAAATTTTGCAT
CCTTTGCCTCCGTTCAAGTATATAAAGTCGGCATGCTTGATAATCTTTCTTTCCATCCTACATTGTTCTAATTATTC
TTATTCTCCTTTATTCTTTCCTAACATACCAAGAAATTAATCTTCTGTCATTCGCTTAAACACTATATCAATA**ATGA**
GGAGGGCTTTTGTAGAAAGAAATACGAACGAAACGAAAATCAGCGTTGCCATCGCTTTGGACAAAGCTCCCTTACCT
GAAGAGTCGAATTTTATTGATGAACTTATAACTTCCAAGCATACAAACCAAAAGGGAGAACAAGTAATCCAAGTAGA
CACGGGAATTGGATTCTTGGATCACATGTATCATGCACTGGCTAAACATGCAGGCTGGAGCTTACGACTTTACTCAA
GAGGTGATTTAATCATCGATGATCATCACACTGCAGAAGATACTGCTATTGCACTTGGTATTGCATTCAAGCAGGCT
ATGAGTAACTTTGCCGGCGTTAAAAGATTTGGACATGCTTATTGTCCACTTGACGAAGCTCTTTCTAGAAGCGTAGT
TGACTTGTCGGGACGGCCCTATGCTGTTATCGATTTGGGATTAAAGCGTGAAAAGGTTGGGGAATTGTCCTGTGAAA
TGATCCCTCACTTACTATATTCCTTTTCGGTAGCAGCTGGAATTACTTTGCATGTTACCTGCTTATATGGTAGTAAT
GACCATCATCGTGCTGAAAGCGCTTTTAAATCTCTGGCTGTTGCCATGCGCGCGGCTACTAGTCTTACTGGAAGTTC
TGAAGTCCCAAGCACGAAGGGAGTGTTGTAATCAGTACTGACAATAAAAAGATTCTTGTTTTCAAGAACTTGTCATT
TGTATAGTTTTTTTATATTGTAGTTGTTCTATTTTAATCAAATGTTAGCGTGATTTATATTTTTTTTCGCCTCGACA
TCATCTGCCCAGATGCGAAGTTAAGTGCGCAGAAAGTAATATCATGCGTCAATCGTATGTGAATGCTGGTCGCTATA
CTGCTGTCGATTCGATACTAACGCCGCCATCCAGT**GCTGTTCGTGTGCGCGTCCTGGG**TGACACCGATTATTTAAAG
CTGCAGCATACGATATATATACATGTGTATATATGTATACCTATGAATGTCAGTAAGTATGTATACGAACAGTATGA
TACTGAAGATGACAAGGTAATGCATCATTCTATACGTGTCATTCTGAACGAGGCGCGCTTTCCTTTTTTCTTTTTGC
TTTTTCTTTTTTTTTCTCTTGAACTCGAGAAAAAAAAATATAAAAGAGATGGAGGAACGGAAAAAGTTAGTTGTGGT
GATAGGTGGCAAGTGGTATTCCGTAAGAACAACAAGAAAAGCATTTCATATTATGGCTGAACTGAGCGAACAAGTGC
AAAATTTAAGCATCAACGACAACAACGAGAATGGTTATGTTCCTCCTCACTTAAGAGGAAAACCAAGAAGTGCCAGA
AATAACAGTAGCAACTACAATAACAACAACGGCGGCTACAACGGTGGCCGTGGCGGTGGCAGCTTCTTTAGCAACAA
CCGTCGTGGTGGTTACGGCAACGGTGGTTTCTTCGGTGGAAACAACGGTGGCAGCAGATCTAACGGCCGTTCTGGTG
GTAGATGGATCGATGGCAAACATGTCCCAGCTCCAAGAAACGAAAAGGCCGAGATCGCCATATTTGGTGTCCCCGAG
GATCCAAATTTCCAATCTTCTGGTATTAACTTCGATAACTACGATGATATTCCAGTGGACGCCTCTGGTAAGGATGT
TCCTGAACCAATCACAGAATTTACCTCACCTCCATTGGACGGATTGTTATTGGAAAACATCAAATTGGCCCGTTTCA
CCAAGCCAACACCTGTGCAAAAATACTCCGTCCCTATCGTTGCCAACGGCAGAGATTTGATGGCCTGTGCGCAGACC
GGTTCTGGTAAGACTGGTGGGTTTTTATTCCCAGTGTTGTCCGAATCATTTAAGACTGGACCATCTCCTCAACCAGA
GTCTCAAGGCTCCTTTTACCAAAGAAAGGCCTACCCAACTGCTGTCATTA
(989 bp 3′ UTR to *HIS3* shown)

NUP188*::*mCherry*::*ADH1(t)*::*prMX*::*CaURA3*::*MX(t)*::*NUP188(t)

ATGGCTACACCTTCATTTGGCAATTCCTCTCCACAATTGACCTTCACTCATGTTGCTAATTTTATGAACGATGCTGC
TGCTGATGTATCCGCTGTGGATGCCAAGCAATTGGCCCAAATAAGACAGTTTTTGAAAGCTAACAAGACAAATCTTA
TCGAGAGTCTGAACACAATAAGGCAGAATGTAACTTCTTCCGGCGATCATAACAAGTTACGCTCTACAATTGCAAAC
TTATTGCAAATAAACGTCGATAATGATCCATTCTTTGCTCAATCTGAGGACCTTTCTCATGCTGTAGAATTCTTTAT
GTCTGAGAGATCATCTAGACTTCATATAGTATACTCACTTCTCGTTAACCCTGACATCGACCTCGAGACATATTCAT
TTATTGACAACGATAGATTCAATGTAGTGGGGAAATTGATTTCTATAATATCATCTGTAATCCAGAACTACGACATT
ATTACAGCTTCATCTTTAGCACATGATTATAATAACGATCAAGACATGTTCACAATTGTCTCATTGGTGCAGTTAAA
AAAATTTTCTGATTTGAAGTTTATCTTGCAGATATTACAAATTTTGAATTTAATGATCCTCAACACAAAAGTACCAG
TAGATATCGTCAATCAATGGTTTCTACAGTATCAAAATCAATTTGTTGAATTTTGCAGAAATATAAATTCAACAGAC
AAGAGTATAGATACCTCGTCACTACAATTGTACAAGTTTCAGAATTTCCAAGACCTCAGTTACCTCTCAGAAACGCT
TATTTCCAGGATTTCATCTTTATTTACTATCACAACTATATTGATACTTGGACTGAACACTTCAATCGCGCAATTTG
ATATTCAGTCACCTTTGTACATGGATACAGAAACTTTCGACACCGTCAATAGCGCTCTAGAGAATGATGTAGCAACC
AATATCGTCAACGAGGACCCCATTTTCCATCCAATGATTCATTACTCCTGGTCATTTATACTTTATTATAGGCGAGC
ATTACAATCGTCGGAATCATTTGATGATTCAGACATAACGAAATTTGCTCTGTTTGCTGAATCACATGATGTTCTTC
AAAAATTGAATACTCTCTCAGAAATTCTATCCTTTGATCCTGTTTACACCACAGTAATAACAGTATTTTTGGAGTTT
TCTTTAAATTTTATTCCTATAACTGCATCTACTTCCCGGGTTTTCGCCAAGATAATTTCAAAGGCACCAGAGCAGTT
CATTGAGAATTTCTTAACTAATGACACTTTTGAGAAAAAAATTGAGCATCATAAAGGCTAAATTGCCCCTATTGAACG
AATCACTGATCCCGCTAATTAATTTAGCCTTAATTGATACCGAGTTTGCCAATTTTGAATTAAAAGATATATGTTCA
TTTGCTGTTACCAAAAGTAGTTTGAACGATTTGGATTATGACTTAATTGCTGATACAATAACAAACTCTTCTTCATC
CTCCGATATTATAGTGCCTGATTTGATAGAGTTGAAGTCTGATTTACTAGTGGCGCCACCCTTAGAAAACGAAAACT
CTAATTGTCTCTTGTCAATACCAAAATCAACAAAGGGAAAAATTCTTACTATCAAACAACAACAACAACAACAACAA
CAACAGAATGGACAACAGCCGCCAACTACTTCCAATTTGATTATTTTCCTTTACAAATTCAATGGTTGGTCTTTGGT
GGGTAGAATATTGCAAAATCTATTACATTCATATATGGAAAAGGGTACACAACTGGATGACTTACAACATGAATTGA
TGATATCCATAATAAAGTTAGTCACTAACGTTGTCGATCCGAAAACCTCGATCGAGAAATCTAGTGAAATTCTGTCA
TATTTATCAAATTCTCTGGATACTTCAGCCAGCACCATAAATGGAGCGTCGATTATCCAAGTAATTTTCGAGATTTT

TGAAATATCACTACAAAGAAAAGATTATACCTCAATTGTACAATGTTGTGAATTTATGACTATGCTGACACCGAATT
ACCTTCATCTTGTTTCTTCTTACTTGAACAAATCGGATTTGTTAGACAAATATGGTAAGACTGGTCTTTCCAATATG
ATACTCGGTTCGGTTGAATTATCTACGGGGGATTACACATTCACTATCCAATTGCTTAAGCTAACAAAAGTCTTTAT
TCGAGAATCTCTCTCATTGAAAAATATCCATATTTCCAAGAGAAGTAAAATTGATATTATTAATAAGTTGATTCTAC
ATGCAATTCATATCTTTGAGAGTTATTACAACTGGAAGTACAACAACTTCTTACAAAAATTCGAAATTGCCTTCCAT
TTGACATTAATTTTTTATGATGTACTTCATGACGTTTTCACCATAAATCCACATCAAAAGGATCAGTTAATCATTTC
CTCGTCAGCAAACAAATTGTTGCAGTTATTTTTGACTCCCATGGATTCCATTGATTTGGCTCCAAATACTTTGACGA
ATATTCTGATTTCACCATTGAATACCACAACAAAAATTTTAGGCGACAAAATTTTGGGTAATTTATATAGCAAGGTA
ATGAATAACTCCTTCAAGCTGTGCACGCTTTTGATCGCTATTCGAGGTAGCAATCGTGATTTAAAGCCAAGCAACCT
GGAAAAATTGCTTTTCATAAATTCATCAAAATTGGTTGACGTTTATACCCTACCAAGTTATGTTCATTTCAAAGTGC
AGATCATAGAGTTGTTAAGCTATTTAGTAGAGGCTCCTTGGAATGACGACTATCCATTTCTGTTATCTTTCCTTGGT
GAAGCAAAGTCAATGGCATTTTTGAAAGAAGTCCTGTCGGACCTGAGCTCTCCAGTGCAAGATTGGAACCTTCTCCG
AAGTTTGTACATTTTTTTTACTACCCTATTAGAAAGTAAACAAGATGGGTTATCTATCCTCTTCTTAACAGGTCAAT
TTGCCTCCAATAAAAAAATTAATGACGAATCCTCCATAGACAAAAAGAGTTCTATATTAACTGTTTTGCAAAAGAAT
TCTTTACTATTAGATTCAACGCCTGAAGAAGTAAGTTGCAAGTTATTAGAAACGATAACATATGTTTTAAACACTTG
GACAAATTCTAAAATATTCATAAAAGATCCTAAATTTGTGAATTCCTTATTGGCTAAGTTGAAGGACTCAAAAAAAT
TATTTCAGAAAAAGGAAAATTTGACTCGTGACGAAACTGTTTCTTTGATTAAGAAATACAAATTGATCTCAAGGATT
GTAGAAATATTTGCGTTGTGTATTTACAACTCCACTGATTCAAACTCGGAAATTTTAAATTTTTTGAATCAGGAAGA
CTTGTTTGAATTAGTTCATCATTTCTTCCAAATTGATGGGTTCAATAAAACTTTTCATGATGAATTAAACTTAAAAT
TTAAAGAGAAATGGCCCAGTTTAGAGCTTCAGTCGTTCCAAAAAATTCCATTATCCAGAATAAACGAAAATGAGAAT
TTCGGTTATGATATTCCGTTATTAGATATAGTTTTAAAAGCTGACCGTAGTTGGAACGAACCAAGTAAGAGTCAGAC
AAATTTTAAGGAGGAAATAACAGACGCTTCATTAAATTTGCAGTATGTCAATTACGAAATATCTACCGCAAAAGCAT
GGGGCGCACTAATCACCACGTTTGTGAAGAGGAGCACTGTTCCGTTAAATGATGGTTTTGTCGACTTGGTAGAGCAT
TTCCTCAAGTTAAATATTGATTTTGGTTCAGATAAACAAATGTTTACCCAAATATATCTGGAAAGAATTGAGTTGTC
GTTTTACATTTTATATTCGTTCAAGTTGTCGGGAAAATTGTTGAAGGAAGAAAAGATAATTGAATTGATGAACAAGA
TTTTTACAATTTTCAAATCTGGTGAGATTGACTTTATTAAAAATATTGGTAAGTCACTAAAAAATAACTTTTATAGA
CCTCTTTTGAGATCTGTCTTAGTTCTTCTCGAGTTAGTCTCGTCAGGAGATCGTTTTATCGAATTAATATCTGATCA
ACTACTGGAATTTTTTGAATTAGTGTTTAGTAAGGGTGTTTATTTGATACTGTCTGAGATATTATGTCAGATCAATA
AATGCTCCACGAGGGGTTTAAGCACCGATCACACCACCCAAATTGTCAACTTGGAGGATAATACTCAGGATTTATTG
TTATTATTATCACTCTTCAAGAAGATCACCAATGTGAATCCCTCTAAAAACTTCAATGTTATTCTAGCATCATCCCT
AAACGAAGTAGGAACTTTGAAAGTTATACTGAACCTATATTCAAGCGCGCATTTGATAAGGATTAACGATGAACCAA
TTCTTGGGCAAATTACTCTGACCTTTATCTCCGAATTGTGTTCTATTGAGCCAATTGCTGCAAAACTCATCAACAGT
GGATTGTATAGCGTTCTATTGGAAAGTCCACTATCTGTTGCAATACAGCAAGGTGATATAAAACCTGAATTTTCGCC
TAGATTGCACAACATTTGGAGCAATGGTTTGTTATCCATTGTTCTACTACTATTAAGTCAGTTCGGTATCAAGGTTT
TACCTGAGACGTGCCTATTTGTTTCATATTTTGGAAAACAAATAAAATCTACAATATATAACTGGGGTGACAACAAG
TTAGCAGTGTCAAGTTCGTTGATAAAGGAAACGAATCAATTGGTTTTATTGCAAAAAATGCTAAACTTGCTAAATTA
CCAAGAATTGTTTATTCAGCCTAAAAACTCTGATGATCAACAAGAAGCTGTAGAATTGGTTATTGGTCTAGATTCTG
AACATGATAAAAAAAGGTTAAGCGCTGCATTAAGCAAGTTTCTAACACATCCAAAATACTTGAATTCAAGAATAATA
CCTACAACTTTAGAAGAGCAACAGCAGTTGGAGGATGAATCAAGCAGATTGGAGTTCGTCAAGGGTATCAGCAGAGA
CATTAAAGCATTACAAGATTCACTATTTAAGGACGTTGTGAGCAAGGGCGAGGAGGATAACATGGCCATCATCAAGG
AGTTCATGCGCTTCAAGGTGCACATGGAGGGCTCCGTGAACGGCCACGAGTTCGAGATCGAGGGCGAGGGCGAGGGC
CGCCCCTACGAGGGCACCCAGACCGCCAAGCTGAAGGTGACCAAGGGTGGCCCCCTGCCCTTCGCCTGGGACATCCT
GTCCCCTCAGTTCATGTACGGCTCCAAGGCCTACGTGAAGCACCCCGCCGACATCCCCGACTACTTGAAGCTGTCCT
TCCCCGAGGGCTTCAAGTGGGAGCGCGTGATGAACTTCGAGGACGGCGGCGTGGTGACCGTGACCCAGGACTCCTCC
CTGCAGGACGGCGAGTTCATCTACAAGGTGAAGCTGCGCGGCACCAACTTCCCCTCCGACGGCCCCGTAATGCAGAA
GAAGACCATGGGCTGGGAGGCCTCCTCCGAGCGGATGTACCCCGAGGACGGCGCCCTGAAGGGCGAGATCAAGCAGA
GGCTGAAGCTGAAGGACGGCGGCCACTACGACGCTGAGGTCAAGACCACCTACAAGGCCAAGAAGCCCGTGCAGCTG
CCCGGCGCCTACAACGTCAACATCAAGTTGGACATCACCTCCCACAACGAGGACTACACCATCGTGGAACAGTACGA
ACGCGCCGAGGGCCGCCACTCCACCGGCGGCATGGACGAGCTGTACAAGTAAGGCGCGCCACTTCTAAATAAGCGAA
TTTCTTATGATTTATGATTTTTATTATTAAATAAGTTATAAAAAAAAATAAGTGTATACAAATTTTAAAGTGACTCTT
AGGTTTTAAAACGAAAATTCTTATTCTTGAGTAACTCTTTCCTGTAGGTCAGGTTGCTTTCTCAGGTATAGTATGAG
GTCGCTCTTATTGACCACACCTCTACCGGCAGATCCGCTAGGGATAACAGGGTAATAT**AGATCT**GTTTAGCTTGCCT
CGTCCCCGCCGGGTCACCCGGCCAGCGACATGGAGGCCCAGAATACCCTCCTTGACAGTCTTGACGTGCGCAGCTCA
GGGGCATGATGTGACTGTCGCCCGTACATTTAGCCCATACATCCCCATGTATAATCATTTGCATCCATACATTTTGA
TGGCCGCACGGCGCGAAGCAAAAATTACGGCTCCTCGCTGCAGACCTGCGAGCAGGGAAACGCTCCCCTCACAGACG
CGTTGAATTGTCCCCACGCCGCGCCCCTGTAGAGAAATATAAAAGGTTAGGATTTGCCACTGAGGTTCTTCTTTCAT
ATACTTCCTTTTAAAATCTTGCTAGGATACAGTTCTCACATCACATCCGAACATAAACAACCATGACAGTCAACACT

AAGACCTATAGTGAGAGAGCAGAAACTCATGCCTCACCAGTAGCACAACGATTATTTCGATTAATGGAACTGAAGAA
AACCAATTTATGTGCATCAATTGATGTTGATACCACTAAGGAATTCCTTGAATTAATTGATAAATTGGGTCCTTATG
TATGCTTAATCAAGACTCATATTGATATAATCAATGATTTTTCCTATGAATCCACTATTGAACCATTATTAGAACTT
TCACGTAAACATCAATTTATGATTTTTGAAGATAGAAAATTTGCTGATATTGGTAATACCGTGAAGAAACAATATAT
TGGTGGAGTTTATAAAATTAGTAGTTGGGCAGATATTACTAATGCTCATGGTGTCACTGGGAATGGAGTAGTTGAAG
GATTAAAACAGGGAGCTAAAGAAACCACCACCAACCAAGAGCCAAGAGGGTTATTGATGTTAGCTGAATTATCATCA
GTGGGATCATTAGCATATGGAGAATATTCTCAAAAAACTGTTGAAATTGCTAAATCCGATAAGGAATTTGTTATTGG
ATTTATTGCCCAACGTGATATGGGTGGACAAGAAGAAGGATTTGATTGGCTTATTATGACACCTGGAGTTGGATTAG
ATGATAAAGGTGATGGATTAGGACAACAATATAGAACTGTTGATGAAGTTGTTAGCACTGGAACTGATATTATCATT
GTTGGTAGAGGATTGTTTGGTAAAGGAAGAGATCCAGATATTGAAGGTAAAAGGTATAGAGATGCTGGTTGGAATGC
TTATTTGAAAAAGACTGGCCAATTATAATCAGTACTGACAATAAAAAGATTCTTGTTTTCAAGAACTTGTCATTTGT
ATAGTTTTTTTATATTGTAGTTGTTCTATTTTAATCAAATGTTAGCGTGATTTATATTTTTTTTCGCCTCGACATCA
TCTGCCCAGATGCGAAGTTAAGTGCGCAGAAAGTAATATCATGCGTCAATCGTATGTGAATGCTGGTCGCTATACTG
CTGTCGATTCGATACTAACGCCGCCATCCAGTCTTATTTTGCAGGTTATGTAAAGCTACATAATATAATAATGAACA
GTGCAAGTGCAAGGGTGGCCCTTTGTTGCTATACTGCGGACTGCGACTGTATGAGCTTTTTCCATCCCAGTTCTATC
CATTGTGTACTTTTTTTTTTCATCGCCTTACGAACTTCAAAAATATGTAAACAATAAGATAAGTAAATTAACCGAAG
ACTCTCATGCTTTGAAAGCGCCATAAGTGCGCGTGTTTGTGCCTTCTGATATGATATCGTATGCCTAAGTTTCCACA
GTTTCGATTGATTTTAGTTTTGTTTTACTTAATATCAATGATACAATGGTCGGTAATCACTTTTTCGCTGGGGTTCT
TTCTTAATGTCTGCATCTTTGCGTACTTCGTATTCTTCAAATCCTTGCCTGATTTACCGAAACCTCAGCCAAGATTT
GTTGATATCGTACCGGAAAGTTCGAATACTGTGGATGTTGATAAAGAATTGAAGTCTGTCGAGGGTTTAATACAAGA
TGGGAACGCGCAAATTGGGAAAGAATTAGAGAGTATTGTTAATTTAATTATTAAAGATTTTGTTCAGCCTTGGTTCA
CAAAAATCGATAAGAATAGTGACGCAGAGTTCCTCAAAGTAATAAAGTGGAGATTATTACAAACGCTCCTAGTGGTG
AAGGACAAGTTGATGAAGAATGATAGTGCCAGCTTGATTGTTCTGAAGCTGTTGCCTATATTTAACAAACATTTCAG
CACTTTTTGTGATGCCAGAGAAGCTGTGCTAAGCGATTTGACTCTTGAAAGGCATAAATCAGCCAACATCGATCTAC
AAATTGCCGTTGAGTTTAATAAAAATTACAAAATACATAAATCATTATCATTGAAGCCGAATGCTCTTCAGAAAGAA
ATTGAAAAATCCATAAGGAAAACTGTAATCGGGCTCCTTCCTCACCTTTTTGATAATGATGAGTTAGATTCATTGCT
TGTTTTCACATTAATGACGGAAGTTCTCACT
(1000 bp of *NUP188* 3′ UTR shown)

GFY-3435, 3436, 3437, 3438, and 3439 were constructed in an identical fashion.

---

pGF-V1220:

pRS425 + sgRNA(u1): *prSNR52::**crRNA**::tracrRNA::**SUP4(t)***

(Cloned into pRS425 vector using BamHI/XhoI restriction sites)

GGATCCTCACTAAAGGGAACAAAAGCTGGAGCTTCTTTGAAAAGATAATGTATGATTATGCTTTCACTCATATTTAT
ACAGAAACTTGATGTTTTCTTTCGAGTATATACAAGGTGATTACATGTACGTTTGAAGTACAACTCTAGATTTTGTA
GTGCCCTCTTGGGCTAGCGGTAAAGGTGCGCATTTTTTCACACCCTACAATGTTCTGTTCAAAAGATTTTGGTCAAA
CGCTGTAGAAGTGAAAGTTGGTGCGCATGTTTCGGCGTTCGAAACTTCTCCGCAGTGAAAGATAAATGATC**CGGTGG**
ACTTCGGCTACGTAGTTTTAGAGCTAGAAATAGCAAGTTAAAATAAGGCTAGTCCGTTATCAACTTGAAAAAGTGGC
ACCGAGTCGGTGGTGC**TTTTTTTGTTTTTTATGTCTTCGAGTCATGTAATTAGTTATGTCACGCCTCGAG**

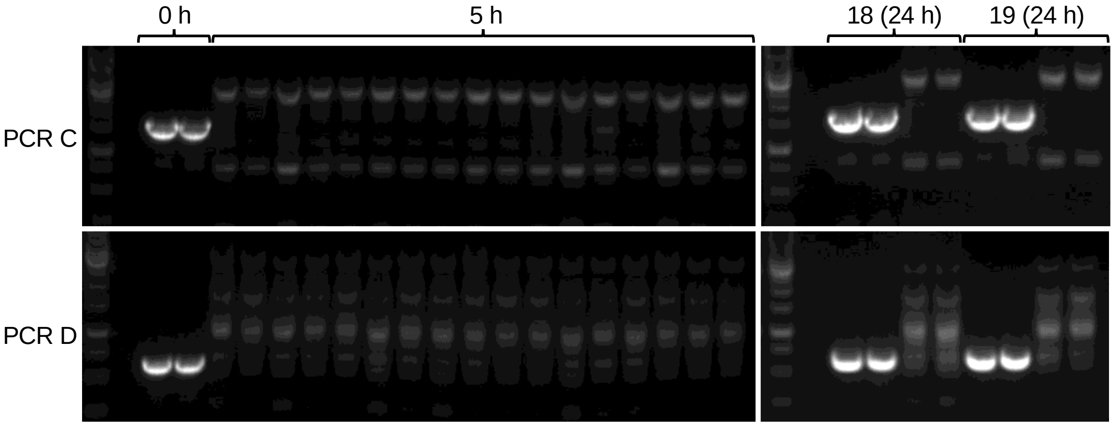

Figure S2. Unmodified DNA gels of diagnostic PCRs used for gene drive analysis. The raw images of the agarose gels used to visualize PCR-C and PCR-D (Fig. 3E) are shown with no modifications except for cropping the image for clarity. *Left*, gels corresponding to the analysis of NLS signals. *Right*, gels corresponding to the analysis of NES signals. Images were collected using the Invitrogen E-Gel^{TM} Imager (ThermoFisher Scientific).

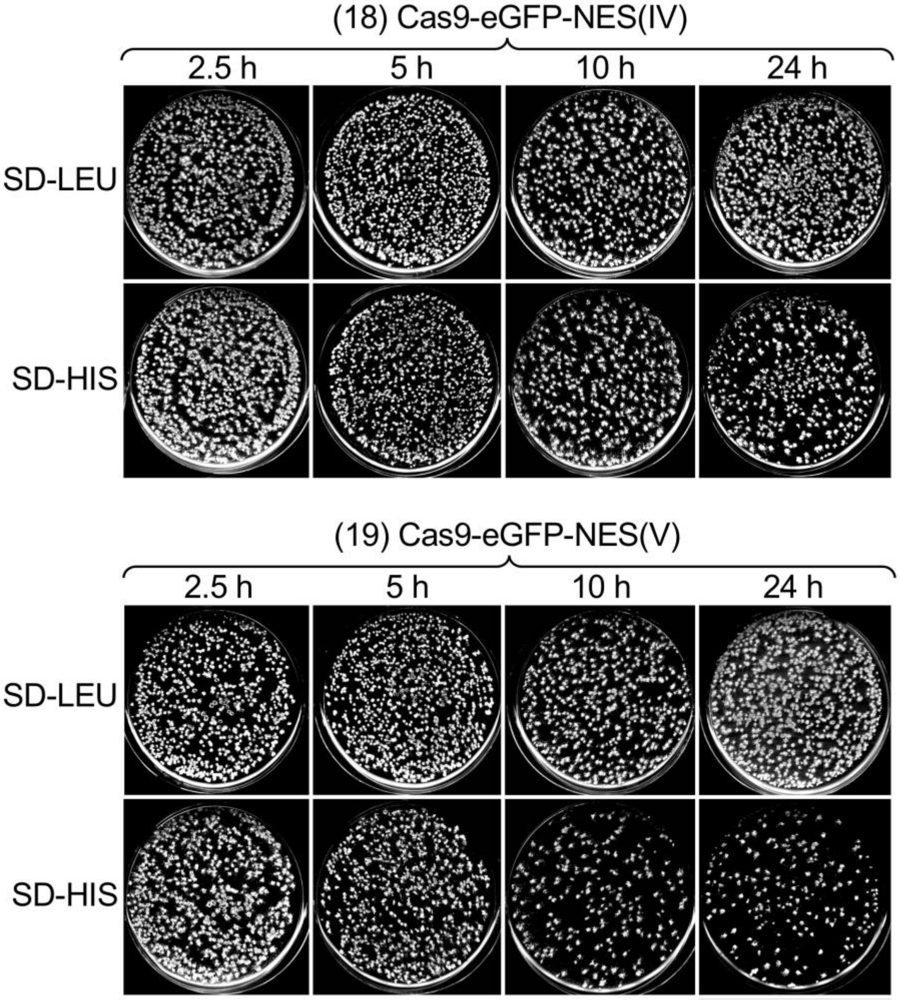

Figure S3. Gene drive agar plate images for Cas9-eGFP-NES fusions. Sample SD-LEU and SD-HIS plates are shown for two constructs (strains 18 and 19 from Fig. 3D) across four galactose induction times (2.5, 5, 10, and 24 h). The sequences for NES(IV) and NES(V) can be found in Fig. 2A.

Table S1. Oligonucleotides used in this study.

**Oligonucleotide Name**	**DNA Sequence (5' to 3')**
F1: prGAL +192 F	GGGGTAATTAATCAGCGAAGCGATGATTTTTG
R1: *SpCas9* Int +373 R	CATCAACGATGTTACCGAAGATTGGATGTC
F2: *SpCas9* Int +2987 F	GCATACTTGAATGCTGTTGTTGGTACTGC
R2: Int GFP R new	CCCGTCATCTTTGAAAAATATAGTTCTTTCCTG
F3: *prHIS3* +196 F	GGCCTCCTCTAGTACACTCTATATTTTTTTATGC
R3: *SHS1(t)* -192 R	GCCATATTTAAATTTATCCCTACAATTATTTGACACTGTTTG
F4: Int pr*CCW12* F	CGTACAAGTATTTCTCAGGAGTAAAAAAACCGTTTG
R4: Int *S. pombe HIS5* R1	CTGCTTGAATGCAATACCAAGTGCAATAGCAG
